# Probing Catalyst Degradation in Metathesis of Internal Olefins: Expanding Access to Amine-Tagged ROMP Polymers

**DOI:** 10.1021/acscatal.3c02729

**Published:** 2023-08-23

**Authors:** Samantha K. Cormier, Deryn E. Fogg

**Affiliations:** † Center for Catalysis Research & Innovation, and Department of Chemistry and Biomolecular Sciences, University of Ottawa, Ottawa, Ontario, Canada K1N 6N5; ‡ Department of Chemistry, University of Bergen, Allégaten 41, N-5007 Bergen, Norway

**Keywords:** olefin metathesis, ROMP, catalyst decomposition, amine, polymer, nucleophile, base

## Abstract

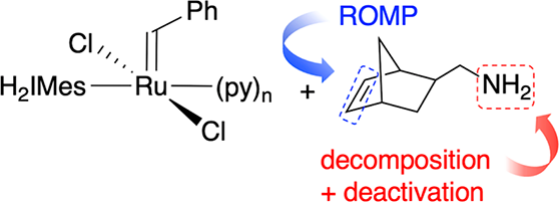

Ruthenium-promoted ring-opening metathesis polymerization (ROMP) offers potentially powerful routes to amine-functionalized polymers with antimicrobial, adhesive, and self-healing properties. However, amines readily degrade the methylidene and unsubstituted ruthenacyclobutane intermediates formed in metathesis of terminal olefins. Examined herein is the relevance of these decomposition pathways to ROMP (i.e., metathesis of *internal* olefins) by the third-generation Grubbs catalyst. Primary alkylamines rapidly quench polymerization via fast adduct formation, followed by nucleophilic abstraction of the propagating alkylidene. Bulkier, Brønsted-basic amines are less aggressive: attack competes only for slow polymerization or strong bases (e.g., DBU). Added HCl limits degradation, as demonstrated by the successful ROMP of an otherwise intractable methylamine monomer.

Amine-functionalized polymers have diverse applications, ranging from CO_2_ uptake,^1,2^ water treatment,^3^ and fuel-cell technologies^4^ to antimicrobial,^5,6^ self-healing,^7,8^ and adhesive^8^ materials (Figure 1a). Improved methods for their production are of keen interest. Cationic and radical polymerization are among the more common synthetic methodologies,^3^ despite limitations arising from water-sensitivity and/or molecular weight control. Ring-opening metathesis polymerization (ROMP), an exceptionally versatile alternative methodology for the assembly of functionalized polymers,^9,10^ is attractive for its operational simplicity. The relative air- and water-stability of widely used ruthenium initiators contributes greatly to ease of production, while the prospect of living ROMP offers control over materials properties.^9^ A clear challenge, however, lies in the ease with which amines degrade the ruthenium metathesis catalysts.^11−15^

**Figure 1:**
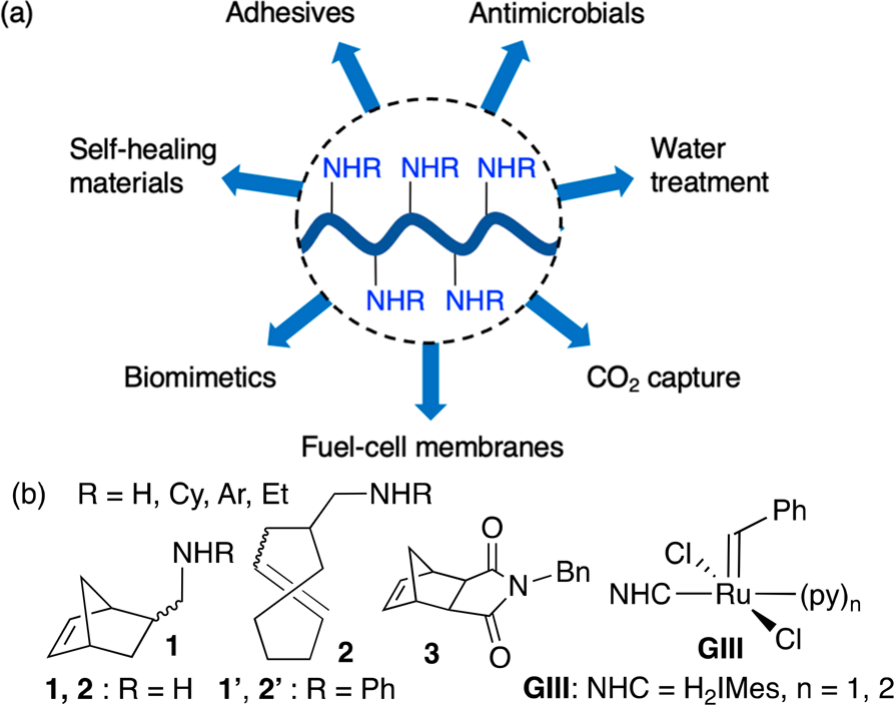
(a) Applications of amine-functionalized polymers. (b) Exemplary monomers and initiator. NHC = *N*-heterocyclic carbene.

Tertiary alkylamines are generally viewed as innocuous in Ru ROMP,^16−18^ despite challenges noted in some reports.^19−21^ Primary and secondary alkylamines, in contrast, are generally serious impediments. In an influential early study, Slugovc documented the adverse impacts of such additives on polymerization rates, yields, and dispersities.^20^ Multiple subsequent reports confirm challenges in ROMP of monomers bearing primary or secondary alkylamines (Figure 1b),^21−25^ although Schafer and co-workers have demonstrated that secondary arylamines (see **1′**/**2′**) can be well-behaved.^23,24^ Protection of primary amines as, e.g., the phthalimide or BOC derivatives^21,25−28^ offers a work-around, but at the price of synthetic efficiency and, in some instances, control over polymer structure.^25^ Deeper understanding of the pathways by which amines impede Ru-promoted ROMP is desirable to devise strategies for the efficient assembly of materials and molecules bearing diverse amine functionalities.

In prior studies focusing on the Ru-catalyzed metathesis of terminal olefins, we established two distinct mechanisms by which amines degrade the active species. Small nucleophilic alkylamines such as NH_2_^*n*^Bu attack **Ru-1** at the methylidene carbon (Scheme 1a, left), generating a [Ru]–CH_2_NH_2_^*n*^Bu species that eliminates NH^*n*^Bu(CH_3_) via a 1,2-proton shift.^12−14,29^ In contrast, tertiary amines function as Brønsted bases, deprotonating the metallacyclobutane **Ru-2** and abstracting a chloride ligand (Scheme 1a, right).^13,14^ The relevance of these pathways to metathesis of *internal* olefins is unexplored. We questioned whether either is plausible in ROMP, given the greater steric encumbrance of the substituted metallacyclobutane and alkylidene species (Scheme 1b). Importantly, however, any steric privileges associated with ROMP reaction manifolds must be balanced against the unforgiving nature of chain-growth polymerization. That is, any perturbation of the initiator or the propagating species will affect polymer chain lengths and dispersities, and hence polymer properties.

**Scheme 1:**
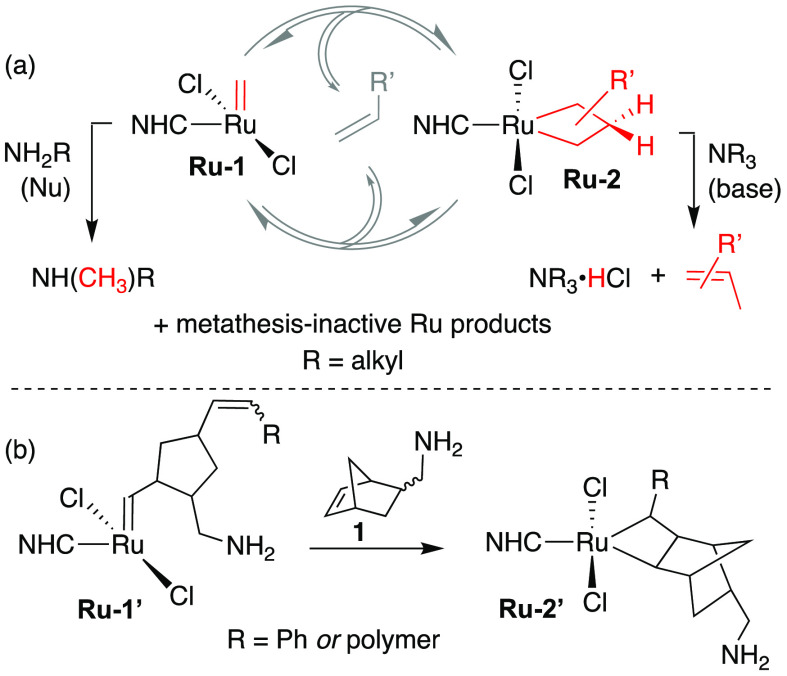
(a) Amine-Induced Degradation of Sterically Accessible Ru Intermediates in Metathesis of 1-Olefins; (b) Potential Steric Protection of Intermediates in ROMP of Exemplary Monomer **1**

Here we set out to determine the impact on ROMP of amines previously shown to decompose the active species in ring-closing and cross-metathesis of 1-olefins (RCM, CM). We find that while weakly basic tertiary alkylamines are tolerated in rapid ROMP reactions, they emerge as problematic when ROMP is slow. Amines that are either sterically undemanding nucleophiles/Lewis bases, or bulky but strong Brønsted bases, represent major hazards.

Our original studies of amine-induced degradation in (R)CM omitted **GIII** (the “third-generation” Grubbs catalyst), as it is little used in the metathesis of terminal olefins.^30,31^ In ROMP, however, **GIII** is one of the preeminent initiators in use.^9^ We thus chose to employ **GIII** to assess the impact on ROMP of amines of varying bulk, basicity, and nucleophilicity^32^ (see **a**–**e**, Scheme 2). Aniline **a**, anticipated to be innocuous,^23,24^ was included to set a baseline for comparison. For the other amines examined, the specific decomposition pathway was established in 1-olefin metathesis: benzylidene abstraction for NH_2_R (**b**, **c**); metallacyclobutane deprotonation for DBU (**d**) and NEt_3_ (**e**).^12−15,33^ Triethylamine was included given the ubiquity of tertiary amines in ROMP polymers, and the conflicting evidence for its detrimental impact noted above.^34^

**Scheme 2:**
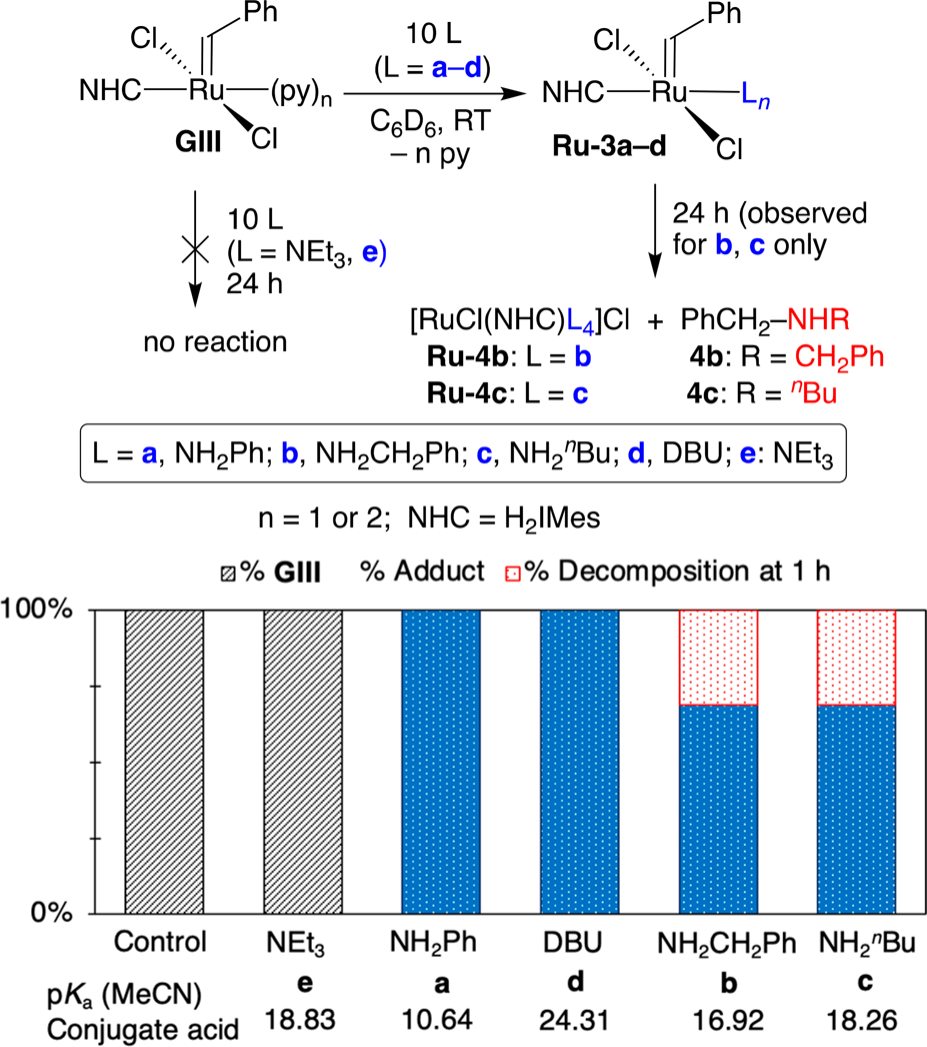
Probing Amine Poisoning and Nucleophilic Attack on GIII by Amines **a**–**e**: Product Distributions at 1 h The (py)_*n*_ notation for **GIII** (*n* = 1, 2) reflects batch-to-batch variation in the number of pyridine ligands.^36,37^ This variability is readily overlooked, as exchange averaging of the ^1^H NMR signals results in a single benzylidene peak.^31^

Initial experiments were carried out in the absence of monomer, to establish which amines degrade **GIII**, and how rapidly. Accordingly, a 10-fold excess of amine was added to solutions of **GIII** in C_6_D_6_ (Scheme 2). The reaction with NH_2_Ph showed quantitative formation of the aniline adduct **Ru-3a** within 15 min, without any apparent color change. This assignment is supported by synthesis of **Ru-3a** on preparative scale (see the Supporting Information). For DBU and the primary alkylamines NH_2_R (R = Bn, ^*n*^Bu), an immediate color change from green to orange was accompanied by quantitative conversion to the known amine adducts **Ru-3b**–**d**.^11−13,35^ No reaction with NEt_3_ was detected even after 24 h, as in a prior study with slower-initiating catalysts.^13^

The aniline and DBU adducts (**Ru-3a** and **Ru-3d**, respectively) were stable over 24 h in solution. In comparison, the alkylamine derivatives **Ru-3b**/**c** underwent ca. 30% loss within 1 h, and complete degradation within 24 h. Benzylidene abstraction was confirmed by observation of the benzylamine derivatives NHR(CH_2_Ph) **4** (74% vs starting **GIII** for **4c**; integration precluded by overlap for **4b**). We conclude that abstraction of the benzylidene ligand—and, by extension, bulkier alkylidenes such as **Ru-1′** (Scheme 1b) or its polymer homologues—is restricted to alkylamines that combine nucleophilicity with steric accessibility. The rapidity of this reaction for **GIII** suggests that initiator degradation may compete with ROMP, particularly for less reactive monomers or at elevated temperatures.

We next considered the question of whether the propagating species are degraded by monomers bearing amine groups, and/or by amines pendant on the polymer chain. We will return to the latter possibility below. To isolate the former, we undertook ROMP of the norbornene succinimide **3** in the presence of equimolar exogenous amine, to simulate an amine-functionalized monomer while precluding intramolecular attack (Scheme 3a). Both exo and endo isomers of **3** were examined, given the differences in amine sensitivity reported for related norbornene stereoisomers.^25^ Control reactions without added amine were complete at 15 min and 24 h for **3-exo** and **3-endo**, respectively.^38^ The corresponding reactions with amine present were assessed at 24 h, to enable maximum conversion in the event that competing formation of the amine adducts **Ru-3** retards but does not terminate polymerization.

**Scheme 3:**
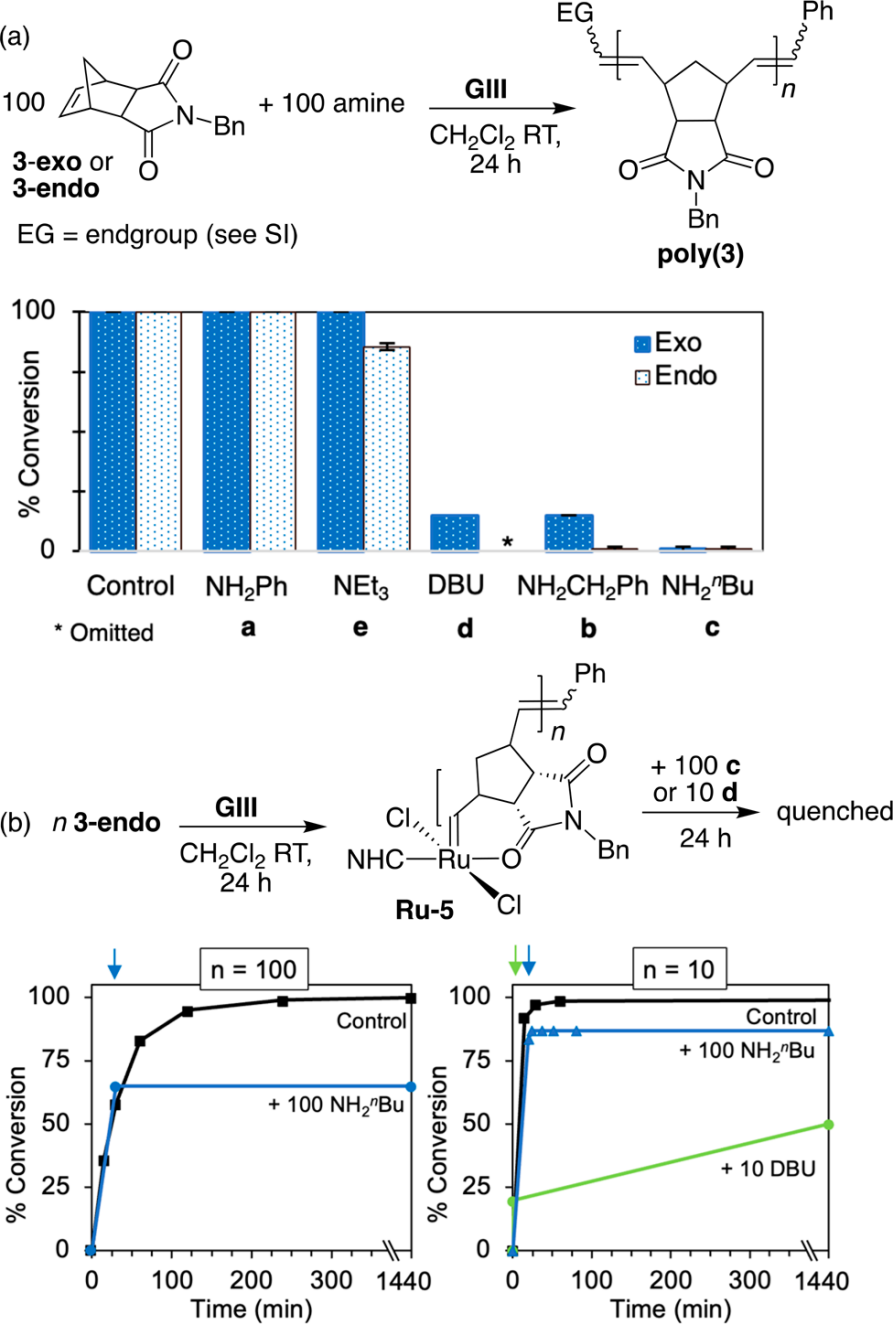
Probing Intermolecular Degradation in ROMP: (a) Amine Present at Outset; (b) Amine Added to Propagating Species Colored arrows denote the time at which amine was added.

In the presence of aniline **a**, ROMP of **3** proceeded to full conversion (Scheme 3a).^23,24^ This tolerance reflects the low nucleophilicity of such aromatic amines: it may be noted that even chelating dianilines are innocuous in RCM.^39^ Added NEt_3_ increases the molecular weights and dispersities of ROMP of **3-exo** slightly (Table S2 and Figure S24), but does not quench polymerization. To assess whether a sterically more accessible metallacyclobutane is more susceptible to deprotonation, we carried out ROMP of 1,5-cycloctadiene in the presence of NEt_3_ (100 equiv; Figure S8). Polymerization was quantitative within 15 min. In contrast, ROMP of **3-endo** (which undergoes ROMP slowly relative to **3-exo**(40)) proceeds to only 86% yield over 24 h under the same conditions. We infer that although deprotonation by NEt_3_ is too slow to compete with propagation for rapid ROMP processes, it can compromise chain-length control if ROMP is slow. For the much stronger Brønsted base DBU, degradation is sufficiently fast that it competes with ROMP of even **3-exo**, and polymer yields drop to just 15%.

Finally, attack by linear primary amines is significantly more aggressive than attack by Brønsted base. Primary alkylamines exert a dramatic inhibiting effect, *n*-butylamine being most deleterious. Complete knockdown of ROMP was observed for both **3**-**exo** and **3-endo**, with ROMP of the slower-reacting **3-endo** again being more significantly affected.

To establish whether decomposition involves nucleophilic attack on the propagating alkylidene or solely uninitiated **GIII**, we repeated the experiment with **3-endo**, but added NH_2_^*n*^Bu (**c**) after ROMP was under way (Scheme 3b, left). A reaction aliquot was removed and quenched with KTp^38^ ca. 2 s prior to amine addition, to assess the impact of added **c** on conversions. As shown in the time–conversion plot, ROMP terminated upon amine addition. Followup experiments in which 100 NH_2_^*n*^Bu was added to a growing oligomer of **3-endo** (<10 repeat units) indicated that initial knockdown involves deactivation via amine binding, with slower decomposition via alkylidene abstraction. Thus, integration of the alkylidene signal vs internal standard indicated only 15% nucleophilic abstraction after 15 min, but 96% after 24 h (Scheme 3b and Figure S6). The slow rate of alkylidene abstraction is striking, given that the known carbonyl-chelated intermediate^40−42^ (see **Ru-5**, Scheme 3b) might be anticipated to facilitate decomposition by “pinning” the alkylidene for attack by the incoming nucleophile.^43^ Importantly, the impact of both nucleophilic degradation and amine poisoning is much enhanced in ROMP of amine-functionalized monomers, where the amine functionality is necessarily present along with the initiator from the outset of reaction. Sterically accessible nucleophilic and Lewis-basic amines clearly compromise controlled polymerization.

A related experiment was conducted with DBU **d** (Scheme 3b, right), using lower proportions of DBU to minimize adduct formation, and higher proportions of **GIII** to enable detection of the DBU·HCl coproduct. DBU was added at 20% conversion (1 min after monomer addition). Ensuing ROMP was dramatically retarded, but the reaction did not terminate immediately. Yields increased by 30% over 24 h, over which time DBU·HCl formed quantitatively, indicating that ROMP and deprotonation of the metallacyclobutane (MCB) are competitive processes. The slow rate of decomposition is unsurprising, given the bulk of both DBU and the trisubstituted MCB, but it is notable that even this sterically encumbered ring is not immune to attack by base.

The discussion above centers on intermolecular reactions with amine. We now consider interactions with amines pendant on the propagating alkylidene, which have a higher probability of encounter with the metal center. To probe this point, we examined metathesis of the notoriously challenging monomer 5-norbornene-2-methylamine **1** (Scheme 4a). Polymers of **1** hold significant potential as water-soluble, haptic, chemically responsive, and pH-responsive materials, but N-protection is essential for their production using Ru initiators.^26,27^ Control experiments indeed showed no polymer on adding **GIII** to 100 equiv of **1** in C_6_D_6_, even after 24 h. Integration against internal standard indicated consumption of <2% **1**, consistent with a stoichiometric inhibition process.

**Scheme 4:**
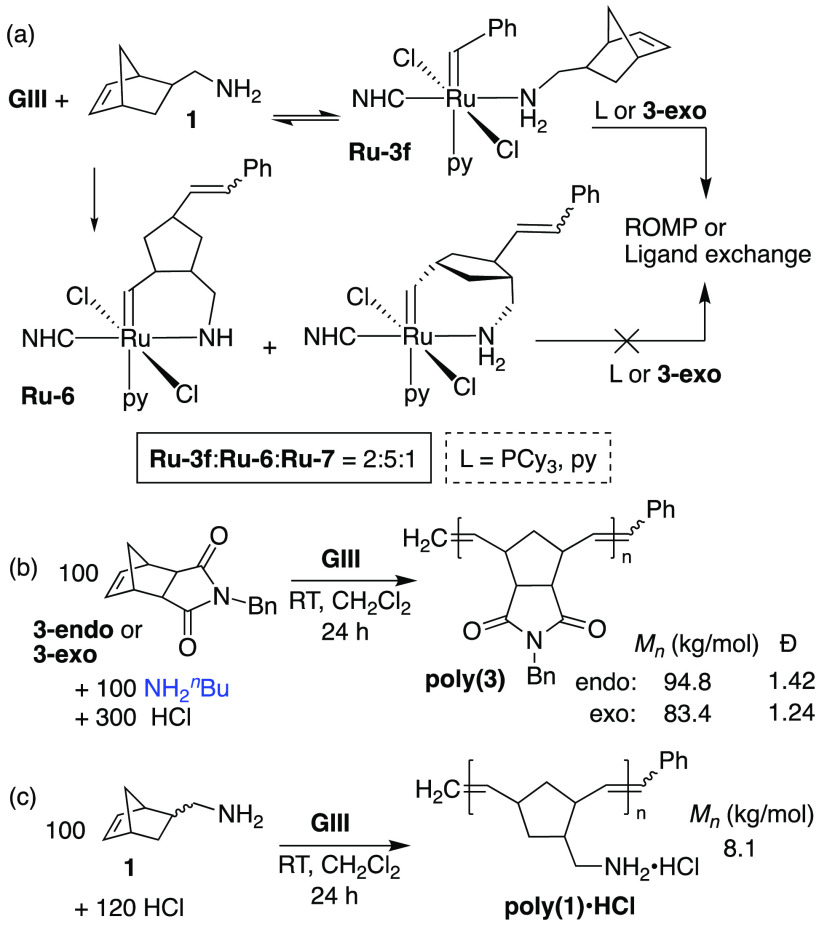
(a) Reaction of GIII with Methylamine Monomer **1**. (b) HCl-Protection as a Mitigating Strategy in ROMP with Exogenous Amine and (c) in ROMP of **1** Reaction (a): no exogenous quenching agent added. Reactions (b), (c): quenched with ethyl vinyl ether (EVE).

To probe the pathways responsible, 1 equiv of **1** was added dropwise to **GIII** in C_6_D_6_.^23^ An immediate color change from green to deep red was observed, with complete conversion of **GIII** into adduct **Ru-3f** and the isomeric amine chelates **Ru-6**/**7** (Scheme 4a) within 10 min. The initial adduct:chelate ratio of 1:3^44,45^ decreased by 10% over the next 24 h, as **Ru-3f** transformed into the chelate complexes (Figure S11). Both formation and decomposition of the chelates are slow, reflecting the low concentration of **1** released in the equilibrium exchange of **GIII** with **Ru-3f**, as well as the multiple pathways open to free **1** (viz, rebinding to Ru, metathesis, or nucleophilic attack). Addition of a further 2 equiv **1** caused complete consumption of **Ru-3f**, and extensive degradation of **Ru-6**/**7** over 24 h (ca. 60%, Figure S13), confirming that metathesis is faster than nucleophilic attack. Finally, experiments involving addition of py, PCy_3_, or **3-exo** to the mixture reveal selective reaction of **Ru-3f**: that is, the κ^2^-amine in **Ru-6**/**7** resists dechelation (Scheme 4a and Figures S14–S16). The stability of the alkylamine chelates contrasts with the comparative lability of related oxygen-bound species.^40−42^

A final set of experiments was directed at mitigation strategies. Acid treatment has been reported as a solution to low productivity in (R)CM of amine-functionalized olefins,^46−49^ in ROMP of pyridine-functionalized monomers^50^ or in the presence of amine donors,^51,52^ and, in a preliminary study, in ROMP of **1** to yield amine·HCl oligomers.^53^ This simple protection strategy offers a potentially attractive alternative to BOC or phthalimide protection, if solubility problems^46^ can be allayed. To probe its efficacy, we repeated ROMP of **3-endo** in the presence of NH_2_^*n*^Bu and a 3-fold excess of HCl (Scheme 4b). This combination of a slowly initiating and hence vulnerable ROMP process and a highly aggressive amine sets a high bar for performance, as indeed illustrated by the annihilation of ROMP seen in Scheme 3b. In sharp contrast, the HCl additive enabled complete polymerization. The **poly(3-endo)** product exhibited lower molecular weights and higher dispersity relative to the control reaction (*M*_n_ 94.8 vs 125.5 kg/mol; *D̵* = 1.42 vs 1.27), as well as a low-molecular-weight tail in the GPC chromatogram (Figure S24). We infer that polymerization and decomposition occur on the same timescale, even in the presence of HCl. That is, HCl enables ROMP, but does not completely inhibit decomposition. Similar behavior in the HBF_4_-enabled RCM of unprotected peptides was attributed to the equilibrium between free and acid-bound amine.^47^ Reduced impacts of HCl on chain lengths and dispersity for **3-exo** are evident in Scheme 4b, as expected from the faster rate of ROMP relative to decomposition.

Even without full quenching of the amine, we considered that this advance holds promise, particularly for applications where strict chain-length control is not essential. We therefore examined the capacity of HCl to impede the aggressive degradation pathways involved in metathesis of **1** (Scheme 4c). ROMP proceeded to full conversion, even for this very challenging monomer. Endgroup analysis of the **poly(1)**·HCl product in D_2_O indicated an average chain length of 50, corresponding to *M*_n_ = 8.1 kDa. Acid protection thus offers a simple, convenient means of achieving ROMP of an otherwise recalcitrant norbornene bearing a primary amine tag.

Amine functionalities are notoriously problematic in olefin metathesis. The foregoing represents the first detailed study of the pathways by which amines hamper the metathesis of internal olefins. While this study focused on amine-functionalized ROMP polymers, these findings hold broader relevance for molecular chemistry.

Our original expectation was that the amine-induced decomposition pathways established in metathesis of terminal olefins—that is, nucleophilic abstraction and base-induced deprotonation—would be largely irrelevant to the sterically more congested species produced by metathesis of internal olefins. This prediction proved false. The negative impact of amines on controlled ROMP is traced to deactivation and decomposition of the propagating species and the initiator. Sterically accessible primary alkylamines pose the greatest hazard, rapidly decomposing the important initiator **GIII**, and terminating ROMP even for highly reactive monomers. Initial knockdown of the propagating alkylidene involves amine binding to the metal, followed by slower, irreversible abstraction of the alkylidene ligand. Deactivation is particularly aggressive, where a pendant amine is positioned for chelation. Bulkier Brønsted bases are also problematic, particularly where basicity is high and/or ROMP is slow.

Addition of HCl was shown to ameliorate these hazards in a model ROMP reaction, with limited perturbation of chain lengths and dispersity. This strategy enables ROMP of an otherwise intractable amine-bearing monomer. While chain-length control is reduced, the capacity to turn off degradation pathways without synthetically cumbersome protecting-group strategies holds promise as a simple, versatile route to amine-functionalized polymers.

## Supporting Information Available

Experimental details, spectra, and tabulated data (PDF)
