# Supplementary material for: Probing Catalyst Degradation in Metathesis of Internal Olefins: Expanding Access to Amine-Tagged ROMP Polymers
Source: ACS Catal. 2023 Aug 23;13(17):11834–40. doi: 10.1021/acscatal.3c02729 (PMC10476157; doi:10.1021/acscatal.3c02729)
Supplement: Supplementary file 1 — cs3c02729_si_001.pdf [file cs3c02729_si_001.pdf]

Probing Catalyst Degradation in Metathesis of Internal Olefins: Expanding Access to Amine-Tagged ROMP Polymers

Samantha K. Cormier[a] and Deryn E. Fogg*[a,b]

[a]Center for Catalysis Research & Innovation, and Department of Chemistry and Biomolecular Sciences, University of Ottawa, ON, Canada, K1N 6N5. [b]Department of Chemistry, University of Bergen, Allégaten 41, N-5007 Bergen, Norway

*Corresponding author: dfogg@uottawa.ca, dfo025@uib.no

Table of Contents

S1. Experimental S2
S2. NMR Spectra. S9
S3. GPC Chromatograms for Polymer Products. S40
S4. Mass Spectrum S41
S5. References S42

S1. Experimental

S1.1 General procedures. All reactions were carried out in an N_2-filled glovebox unless otherwise noted. HPLC-grade CH_2Cl_2 was dried and degassed with a Glass Contour solvent purification system and stored under N_2 over 4 Å molecular sieves for at least 24 h prior to use, to attain water content of ≤4 ppm (confirmed by Karl-Fischer titration). Initiator **GIII**[1] and monomers **3-exo**[2] and **3-endo**[2] were prepared by literature methods. C_6D_6 (Cambridge Isotopes), *n*-butylamine (Alfa Aesar, 99%), benzylamine (Acros Organics, 99%), triethylamine (Sigma-Aldrich, > 99%), 1,8-diazabicyclo[5.4.0]undec-7-ene (DBU, Acros, 98%), and aniline (Sigma-Aldrich, ≥99.5%), were freeze-pump-thaw degassed (4×) and stored under N_2 in the glovebox (C_6D_6 stored over 4 Å sieves 12 h before use). Ethereal HCl (2.0 M, Sigma-Aldrich), pyridine (Sigma-Aldrich, 99.8%), PCy_3 (Strem, 97%), ethyl vinyl ether (contains 0.1% KOH as stabilizer, 99%, Sigma-Aldrich), 5-norbornene-2-methylamine (**1**, TCI, mixture of isomers, 98.0%), 1,5-cyclooctadiene (COD, Sigma-Aldrich, >99%), $CDCl_3$ (Cambridge Isotopes), 1,3,5-trimethoxybenzene (TMB, TCI, 98%), dimethyl terephthalate (DMT, Sigma-Aldrich, 99%), anthracene (Sigma-Aldrich, 96%), and potassium trispyrazolyl borate (KTp, Sigma-Aldrich, 98%) were used as received.

NMR spectra were recorded on Bruker Avance 300, Avance II 300, Avance II 400, Avance III 500, and Avance III 600 MHz NMR spectrometers at 25.0 ±0.5 °C. Chemical shifts (ppm) are referenced to the residual proton of the deuterated solvent. Polymers were analyzed by gel permeation chromatography (GPC) using a multi-detector Malvern OMNISEC GPC equipped with an OMNISEC Resolve pump, an autosampler (CHR7100), two T6000M columns, and an OMNISEC Reveal (CHR6000) differential refractive index, a diode-array UV/Vis, and a Viscotek SEC-MALS 20 multi-angle light-scattering detector. HPLC grade THF (Caledon Laboratory Chemicals) was used as the mobile phase (flow rate 1.0 mL min^{-1}; 30 °C). Triple-detection calibration was performed on the instrument using a narrow-MW polystyrene (Malvern). Samples were dissolved in HPLC grade THF at a concentration of 2.0 mg/mL, and filtered through a syringe filter with 0.2 μM PTFE membrane (VWR international) prior to analysis. Values of dn/dc for samples were determined on-line using the OMNISEC software, which assumes 100% of the injected sample mass elutes from the column. Values for polymers of **3-endo**: 0.025; for **3-exo** polymers: 0.072. Number-average molecular weights (M_n) and dispersities (Đ $=M_w/M_n$) were determined using the OMNISEC software.

Electrospray mass spectrometric analysis was carried out by the John Holmes Mass Spectrometry Facility (University of Ottawa) using a Waters Micromass Q-TOF I mass spectrometer. Ca. 30 μg/mL solutions were injected at a flow rate of 50 μL/min, nebulized using 70 psi N_2 at 200 ºC. The capillary and cone voltages were 3.5 kV and 40 kV respectively and the source was heated to 100 ºC.

Mixing in long-duration NMR-tube experiments was affected by affixing the NMR tube to the rotor spindle of a repurposed rotary evaporator.

S1.2 Measuring T_1 relaxation times for monomers 1 and 3. To minimize inaccuracies in conversion measurements arising from the impact of viscosity changes on relaxation time,[3] qNMR analysis was performed with $5 \times T_1$ for the slowest-relaxing signal of interest (Table S1). The standard 0.01-sec longitudinal relaxation delay (d1) proved insufficient for complete relaxation of the olefinic protons of monomers **1** and **3**, and the appropriate relaxation delay was therefore assessed by measuring T_1 values by the inversion-recovery method in $CDCl_3$.[4]

Table S1. Summary of Relaxation Delay Parameters for Quantitative NMR Analysis

Monomer	Measured T_1 (olefinic signals)	d1 for qNMR
**1**	24 sec	122 sec
**3-endo**	3.4 sec	16.8 sec

S1.3 Representative procedure for ROMP of norbornene monomer 3. Solid **3-endo** (13 mg, 0.05 mmol; 100 equiv vs Ru) was added to a solution of ca. 10 mg anthracene (internal standard (IS) for NMR analysis) in 964 μL CH_2Cl_2. The initial ratio of monomer to IS was assessed by removing an aliquot for ^1H NMR analysis. Green **GIII** (36 μL of a 10 mg/mL stock solution in CH_2Cl_2, 0.5 μmol) was then added rapidly to the stirred solution. The reaction was stirred at RT for 24 h, at which point an aliquot was removed and quenched with KTp (10 mg/mL in THF; 10 equiv vs starting Ru), which effects knockdown much more rapidly than ethyl vinyl ether[5] (see Section 1.8). Conversions were assessed by ^1H NMR analysis, from the disappearance of the characteristic olefinic signal for **3-endo** (5.89 ppm, $CDCl_3$). ^1H NMR data for poly(**3**) agree with values previously reported.[2,6] The endgroup resulting from KTp quenching is depicted in Figure S1.

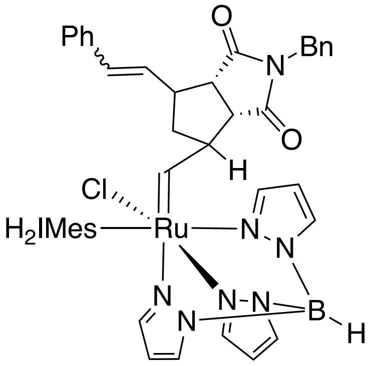

Figure S1. Expected endgroup for polymerizations quenched with KTp, proposed by analogy to the established reaction of **GI** ($RuCl_2(PCy_3)_2(=CHPh)$) with KTp.[7]

Assessing impact of amines on ROMP of 3: As for **3-endo**, but adding 0.05 mmol amine (equimolar with monomer) from a stock solution in CH_2Cl_2 prior to adding **GIII**. See Figure S5 for a representative spectrum showing quantitation.

Impact of HCl and $NH_2{}^n$Bu. As for **3-endo** with $NH_2{}^n$Bu, but with 0.15 mmol HCl (2.0 M in diethyl ether, 120 equiv vs Ru) added prior to **GIII** and quenched with ethyl vinyl ether.

Isolation of polymers. All polymers synthesized for the purpose of structural analysis (as opposed to mechanistic / kinetics analysis) were quenched with ethyl vinyl ether to remove the Ru endgroup from the polymer. Reaction mixtures were concentrated and cold MeOH (ca. 1 mL) was added to afford the polymer as a beige solid, which was filtered off, rinsed with MeOH, dried and redissolved in THF for analysis. See Table S2 for polymer characterization data and Figure S24 for GPC traces.

S1.4 Representative procedure for ROMP of COD. COD (22 µL of a 206 mg/mL stock solution in C_6D_6, 0.043 mmol; 100 equiv) was added to solution of NEt_3 (20 µL of a 220 mg/mL stock solution in CD_2Cl_2, 0.043 mmol; 100 equiv) and ca. 1 mg anthracene in 787 µL CD_2Cl_2 in a J-Young NMR tube. The tube was shaken vigorously to mix, and the initial ratio of COD to IS was measured by ^{1}H NMR analysis. A solution of **GIII** (20 µL of a 15 mg/mL stock solution in C_6D_6, 0.5 µmol) was added and the NMR tube was inverted continuously to mix as described in the General Experimental. ^{1}H NMR analysis at 15 min indicated complete disappearance of the olefinic signals for COD at 5.58 ppm (Figure S8).

S1.5 Representative procedure for ROMP of methylamine monomer 1. 5-Norbornene-2-methylamine **1** (60 µL of a 103 mg/mL stock solution in CH_2Cl_2, 0.05 mmol; 100 equiv vs Ru) was added to a solution of ca. 10 mg anthracene (internal standard, IS) in 877 µL CH_2Cl_2. Addition of ethereal HCl (30 µL of a 2.0 M solution; 0.15 mmol, 1.2 equiv vs **1**) caused a white precipitate to form. An aliquot (10 drops) was removed by pipette to establish the initial ratio of IS to monomer. Green **GIII** (33 µL of a 10 mg/mL stock solution in CH_2Cl_2, 0.5 µmol) was added rapidly, and the suspension was stirred for 24 h. Aliquots were quenched with 5 drops of ethyl vinyl ether and conversions were assessed as above, from disappearance of the characteristic olefinic ^{1}H NMR signal for **1** (6.21 and 6.07 ppm, $CDCl_3$; see Figure S9). The polymer was filtered off and dissolved in D_2O for endgroup analysis. A homogeneous solution was obtained on use of MeOH as the solvent for ROMP, but strong H-bonding precluded endgroup analysis.

^{1}H NMR (300 MHz, D_2O) δ 5.63-5.08 (olefin C*H*, br), 3.24-2.63 (br), 2.59-2.39 (br), 2.34-2.13 (br), 2.07-1.77 (br), 1.72-1.49 (br), 1.27-0.89 (br). The breadth of the signals hampered assignment and integration. NMR endgroup analysis indicates a degree of polymerization of 50 for **poly(1)•**HCl: see Figure S10.

S1.6 Procedure for ROMP of succinimide monomer 3-endo with delayed addition of DBU. Solid **3-endo** (22 mg, 0.088 mmol; 10 equiv) was added to a solution of ca. 10 mg TMB (IS) in 455 µL CH_2Cl_2. After removing an aliquot (5 drops) for NMR analysis to establish the initial ratio of IS to monomer, green **GIII** (636 µL of a 10 mg/mL stock solution in CH_2Cl_2, 8.8 µmol; 1 equiv vs **3-endo**) was added rapidly to the monomer solution. A colour change from green to brown was complete in <1 min. An aliquot of 5 drops was removed and quenched with KTp for analysis, immediately prior to adding DBU (493 µL of a 27 mg/mL stock solution in C_6D_6, 0.088 mmol; 10 equiv) to the reaction. DBU addition effected an immediate colour change from green to red. ^{1}H

NMR analysis was performed to measure conversion based on the characteristic olefinic protons at 5.89 ppm ($CDCl_3$). ^{1}H NMR data for DBU•HCl agree with values reported[6] (see Figure S6).

S1.7 Procedure for ROMP of succinimide monomer 3-endo with delayed addition of $NH_2{}^n$Bu. As in S1.3 above, with the following modifications. An aliquot was removed and quenched with KTp solution (10 mg/mL in THF; 16 equiv vs starting Ru) at 30 min. Within ca. 2 sec, $NH_2{}^n$Bu (281 µL of a 13 mg/mL stock solution in CH_2Cl_2; 0.05 mmol, 100 equiv vs Ru) was injected into the stirred reaction. Conversions were assessed from disappearance of the characteristic olefinic ^{1}H NMR signal for **3-endo** (5.89 ppm, $CDCl_3$). The putative endgroup for polymers quenched by $NH_2{}^n$Bu is shown in Figure S2.

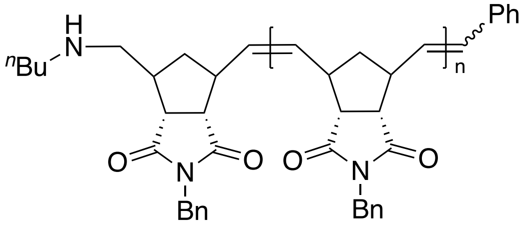

Figure S2. Putative endgroup for ROMP quenched by alkylidene abstraction with $NH_2{}^n$Bu.

S1.8 Assessing extent of nucleophilic abstraction from oligo(3-endo) by $NH_2{}^n$Bu. To an NMR tube with a screw-cap equipped with a rubber septum was added 0.5 mL of a 10 mg/mL solution of **GIII** (5 mg, 6.9 µmol, 1.0 equiv) and ca. 1 mg DMT. The initial ratio of DMT to **GIII** was established by ^{1}H NMR analysis (Figure S7). The solution of **GIII** was then poured into a vial with a stir bar and stirred rapidly while adding monomer **3-endo** (18 mg, 0.071 mmol, 10.4 equiv) as a solution in ca. 0.3 mL CD_2Cl_2. At 22 min, no further **GIII** remained (^{1}H NMR) and 100 $NH_2{}^n$Bu (68 µL, 0.69 mmol) was injected. Conversions of monomer and loss of alkylidene signals were assessed for the first hour and again 24 h after adding $NH_2{}^n$Bu (Figure S3). No further consumption of **3-endo** was observed, but alkylidene abstraction continued over 24 h.

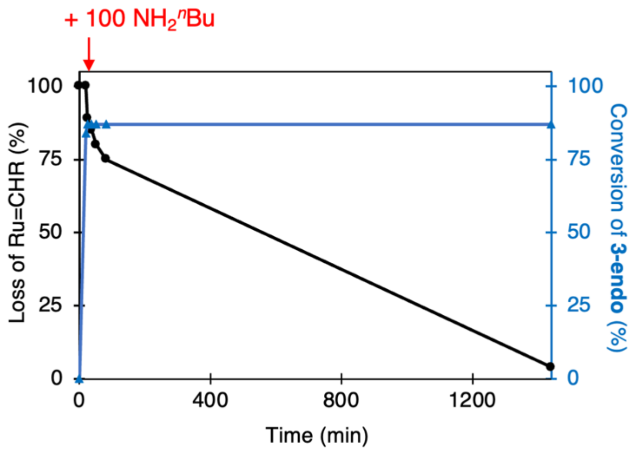

Figure S3. Assessing timescale of quenching vs alkylidene abstraction by $NH_2{}^n$Bu during ROMP of **3-endo**. Lines are provided as a visual guide, not curve fits.

S1.9 Assessing efficacy of KTp in quenching ROMP. Solid **3-exo** (13 mg, 0.05 mmol; 100 equiv) was added to DMT (ca. 2 mg) in 1 mL $CDCl_3$. Green **GIII** (33 μL of a 11 mg/mL stock solution in CH_2Cl_2, 0.5 μmol); 1 equiv vs **3-exo**) was added rapidly while stirring. After 30 sec, the solution was split into two separate J-Young NMR tubes, one containing 27 μL KTp solution (46 mg/mL in THF, 5 μmol, ca. 20 equiv relative to Ru). The NMR tubes were inverted to mix prior to ^{1}H NMR analysis. The two reactions were analyzed as rapidly as possible (at 8 min for the KTp-quenched solution, 14 min for the control: Figure S4). The control reaction reached full conversion by 14 min. The reaction with KTp present showed <1% increase over 24 h.

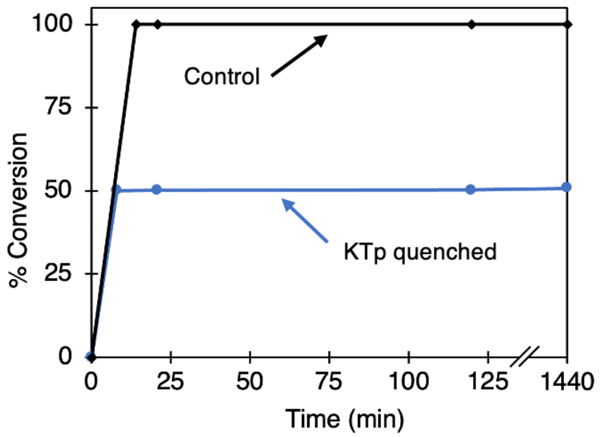

Figure S4. Rate plots confirming quenching of ROMP of **3-exo** by KTp. Curves show quenching at 30 sec vs unquenched control.

S1.10 In situ generation of norbornene-methylamine complexes Ru-3f, Ru-6, Ru-7. Dropwise addition of a solution of **1** (48 μL of a 70 mg/mL stock solution in C_6D_6; 0.027 mmol; 1 equiv) over 5 min to a vigorously stirred solution of **GIII** (20 mg, 0.027 mmol) in 0.5 mL C_6D_6 caused an immediate colour change to red. Complete conversion of **GIII** was confirmed by NMR analysis within 10 min (see Figure S11). For stability of these species in solution, see Figure S12.

^{1}H NMR (500 MHz, C_6D_6; alkylidene signals only): δ 19.70-19.63 (m), **Ru-3f**; 19.20 (t, $^3J_{HH}$ = 3.6 Hz), **Ru-6**; 18.85 (d, $^3J_{HH}$ = 5.5 Hz), **Ru-7**. See Scheme S1 for origin of stereochemistry of **Ru-6/7**. For 2D NMR characterization, see Figures S17-19.

ESI-MS (THF): Calcd for $C_{36}H_{44}Cl_2N_3Ru$ ($[M–pyH]^+$), *m/z* 690.19. Found: *m/z* 690.15 (Figure S25).

Scheme S1. Origin of isomeric chelate complexes **Ru-6** and **Ru-7** in the orientation of approach of **1** to **GIII** in the cycloaddition step.

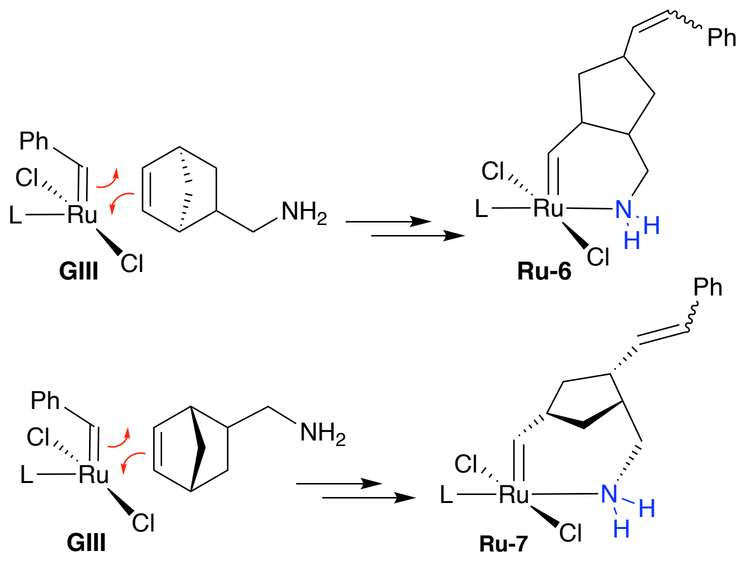

S1.11 Assessing lability of monodentate vs chelated amine ligand 1.

Reaction of Ru-3f and Ru-6/7 with PCy_3. A red solution of **Ru-3f**, **Ru-6,** and **Ru-7** generated as above (18 mg **GIII**, 0.025 mmol) was transferred to a screw-cap NMR tube. A gas-tight syringe was filled with a solution of PCy_3 (39 µL of a 192 mg/mL stock solution in C_6D_6, 0.027 mmol; 1.1 equiv), and the needle tip was inserted into the edge of a rubber septum to seal against air. Both were removed to the NMR room, where the PCy_3 solution was rapidly injected, the pierced NMR-tube cap was sealed with Parafilm, the tube was inverted to mix, and NMR analysis was carried out within 5 min of injection. Mixing was maintained prior to analysis at 1 h and 24 h as described in the General Procedures. For data, see Figure S14.

Reaction of Ru-6/7 and Ru-3f with norbornene monomer 3-exo. A red solution of **Ru-6, Ru-7** and **Ru-3f** was generated as in **S1.9** (18 mg **GIII**, 0.025 mmol), and solid white **3-exo** was added (12 mg, 0.05 mmol, 2.0 equiv). The solution was mixed and analyzed at 24 h. For ^{1}H NMR spectrum, see Figure S15.

Reaction of Ru-6/7 and Ru-3f with methylamine monomer 1. As for **3-exo** (10 mg **GIII**, 0.0138 mmol), but adding **1** (242 µL, 0.028 mmol, 2.0 equiv) instead of **3-exo** (Figure S13).

Reaction of Ru-6/7 and Ru-3f with pyridine. As for **3-exo** (10 mg **GIII**, 0.0138 mmol), but adding pyridine (50 µL, 0.62 mmol, 45 equiv) instead of **3-exo** (Figure S16).

S1.15 Representative procedure for ^{1}H NMR experiments of GIII with amines (10 equiv). Solid green **GIII** (10 mg, 0.014 mmol) was dissolved in ca. 0.5 mL C_6D_6 with ca. 2 mg TMB (IS) and transferred to a J-Young NMR tube. The initial ratio of catalyst to IS was measured (^{1}H NMR), after which the tube was returned to the glovebox and $NH_2{}^n$Bu (10 mg, 0.14 mmol, 10 equiv) was

added. Mixing was conducted as above. ^{1}H NMR spectra were measured at 1 and 24 h (for representative spectra, see Figures S20-23).

S1.16 Synthesis of $RuCl_2(H_2IMes)(NH_2Ph)(=CHPh)$, Ru-3a. NH_2Ph (138 mg, 1.5 mmol, 10 equiv) was added to a green solution of **GIII** (108 mg, 0.15 mmol) in C_6H_6 (5 mL). NMR analysis of an aliquot after 15 min at RT confirmed complete conversion of **GIII**, although no colour change was apparent. The solution was concentrated to ca. 0.5 mL, treated with hexanes (10 mL), and cooled to –35 °C for 20 min to precipitate the product. The green powder was filtered off, washed with hexanes (3 × 3 mL) to remove free NH_2Ph, and dried under vacuum overnight. Also present was residual hexanes (ca. 10%), the proportion of which was unaffected by prolonged drying under vacuum (50 mTorr). Yield: 94 mg (94%, corrected for hexanes solvate).

^{1}H NMR (300 MHz, C_7D_8, –30 °C): δ 19.48 (s, 1H, [Ru]=C*H*), 8.30 (s, 2 H, NH_2Ph *o*-C*H*), 7.97 (s, 2H, Ph *o*-C*H*), 7.18 (overlap with NMR solvent, Ph *p*-C*H*), 6.88 (overlap with NMR solvent, Ph *m*-C*H*), 6.61 (s, 2H, Mes-C*H*), 6.37 (s, 1H, NH_2Ph *p*-C*H*), 6.07 (s, 2H, NH_2Ph *m*-C*H*), 3.5–3.2 (m, 4H, H_2IMes–CH_2), 2.88 (s, 6H, Mes, *p*-CH_3), 2.45 (s, 6H, Mes, *o*-CH_3), 2.18 (s, overlap with NMR solvent, Mes, *o*-CH_3). Also present: hexanes, 0.88 (CH_3), 1.22 (CH_2) ^{13}C{^{1}H} NMR (75.4 MHz, C_7D_8, –30 °C): δ 312.8 ([Ru]=*C*H), 218.1 (C_{NHC}), 152.6 (NH_2Ph, C_i), 151.7 (Ph, C_i), 139.9 (Mes), 138.3 (Mes), 137.9 (Mes), 137.7 (Mes), 137.0 (Mes), 135.7 (Mes), 130.6 (Ph, *o*-*C*H), 129.7, (overlap with NMR solvent, Mes, *C*H), 129.5 (overlap with NMR solvent, Ph, *m*-*C*H), 128.4 (overlap with NMR solvent, Ph, *p*-*C*H), 128.0 (NH_2Ph, *C*H), 123.0 (NH_2Ph, *C*H), 115.8 (NH_2Ph, C*H*), 51.3 (NHC, *C*H_2), 50.3 (NHC, *C*H_2), 20.8 (overlap with NMR solvent, Mes, C-*C*H_3), 20.7 (overlap with NMR solvent, Mes, C-*C*H_3), 18.7 (Mes, C-*C*H_3). IR (ATR, cm^{-1}), ν(N-H): 3364 (w), 3294 (w). Combustion analysis was not undertaken, given the recalcitrant solvation noted above.

S1.17 Synthesis of $RuCl_2(H_2IMes)(NH_2CH_2Ph)(=CHPh)$, Ru-3b. To a stirred green solution of **GIII** (40 mg, 0.055 mmol) in 5 mL C_6H_6 was added NH_2CH_2Ph (59 mg, 0.55 mmol, 10 equiv). A colour change from green to red occurred within 15 min. The solution was concentrated to ca. 0.1 mL, and 5 mL hexanes was added to precipitate **Ru-3b**. The yellow solid was filtered off, washed with 3×1 mL hexanes, and dried under vacuum. Yield: 27 mg (68%).

^{1}H NMR (600 MHz, C_6D_6): δ 19.64 (s, 1H, [Ru]=C*H*), δ 8.30 (d, 2H, $^3J_{HH}$ = 7.8 Hz, *o*-Ph), δ 7.29 (t, 1H, $^3J_{HH}$ = 7.3 Hz, *p*-Ph), δ 7.05 (t, overlapping, $^3J_{HH}$ = 7.8 Hz), δ 7.00-6.78 (br, overlapping), δ 6.49 (br, s, 2H, H_2IMes Ar), δ 3.43 (br, s, overlapping, NH_2CH_2Ph CH_2), 3.34 (br, s, overlapping, H_2IMes CH_2), δ 2.82 (s, 6H, H_2IMes *o*-CH_3), δ 2.32 (s, 6H, H_2IMes *o*-CH_3), δ 2.02 (s, 6H, H_2IMes *p*-CH_3). Signals assigned by analogy to known **GIII**[1] and **GII**-pyrrolidine adduct.[8] Exchange of NH_2CH_2Ph precluded 2D characterization (cross-peaks not observed).

S2. NMR Spectra.

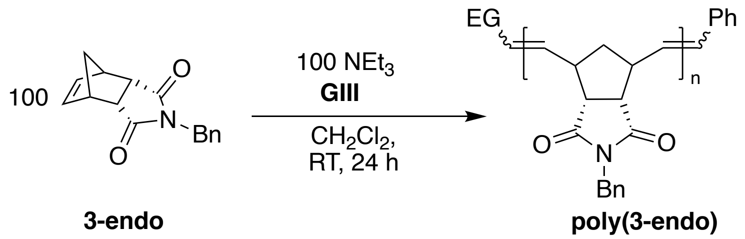

(a) Ratio of starting **3-endo** to anthracene (internal standard) at t = 0 h

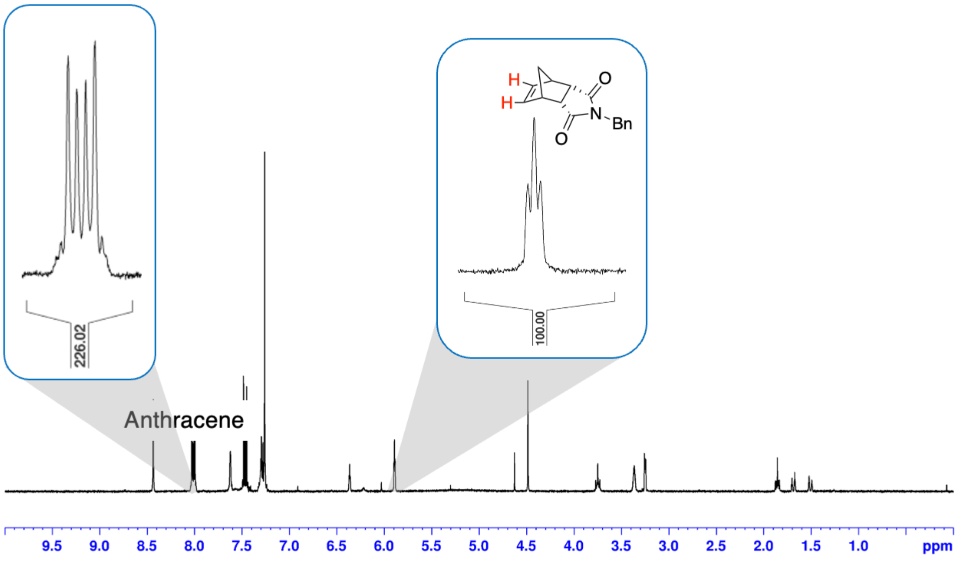

(b) t = 24 h

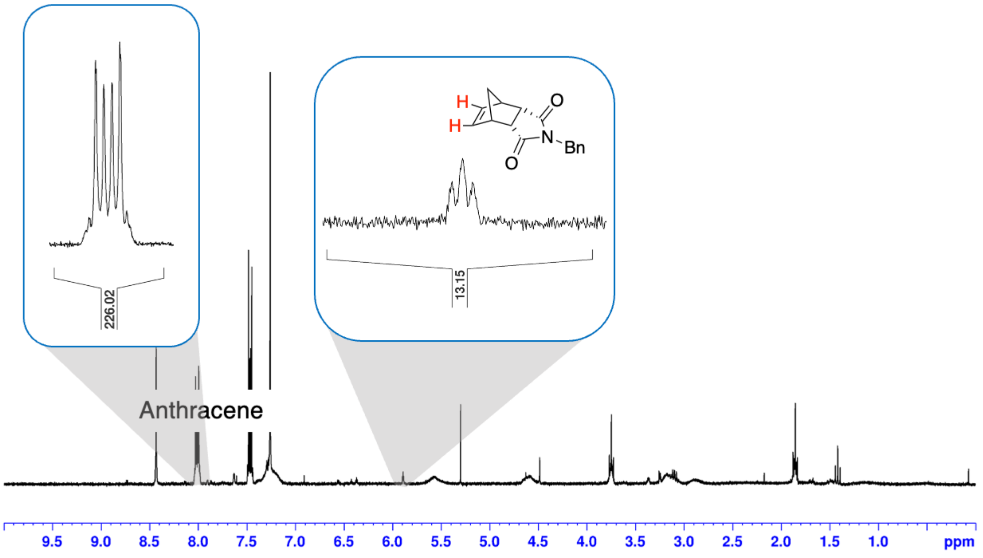

Figure S5. Representative ^{1}H NMR spectra (300 MHz, $CDCl_3$) showing quantification of ROMP conversions with **3-endo.** (a) Before adding initiator. (b) 24 h after adding initiator.

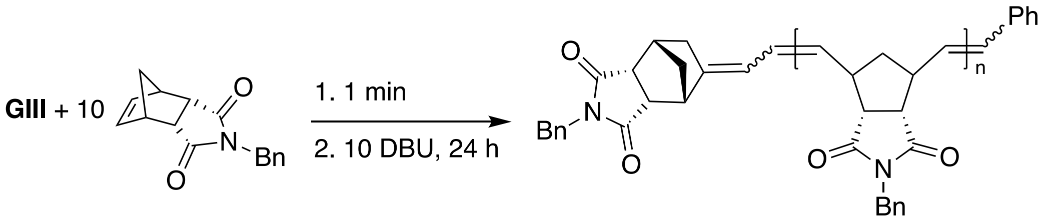

(a) Initial ratio of starting **3-endo** to TMB (internal standard)

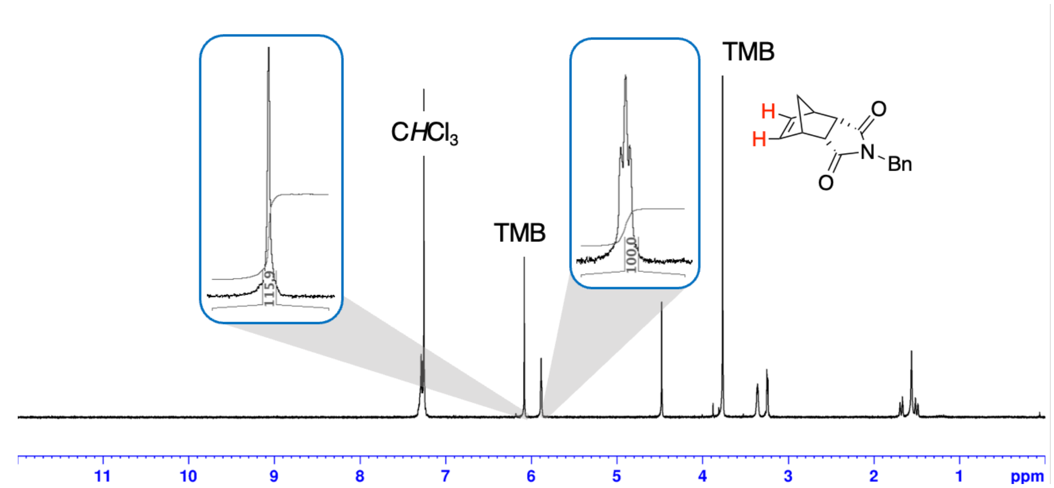

(b) t = 1 min

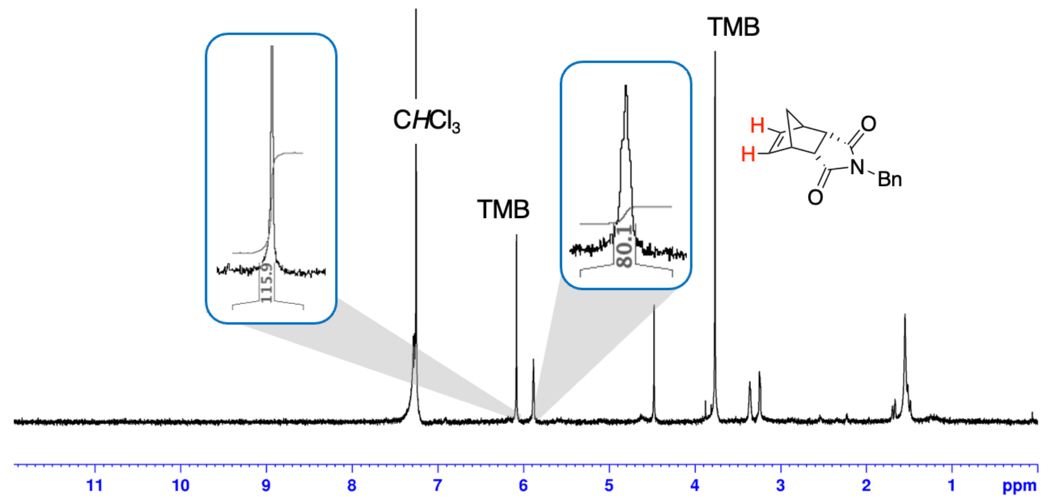

(Continues on next page)

(c) t = 24 h

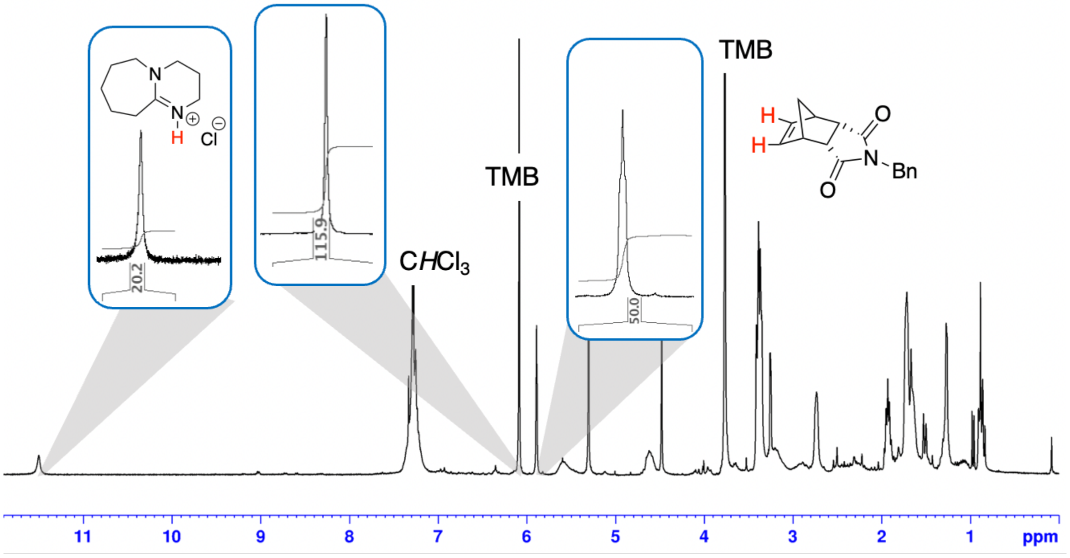

Figure S6. Representative quantitation of conversions in ROMP of **3-endo**. (a) ^{1}H NMR spectrum (300 MHz, $CDCl_3$) prior to adding initiator **GIII**. (b) Spectrum 1 min after adding **GIII** and immediately prior to adding DBU. (c) Spectrum 24 h later, showing remaining signals for monomer.

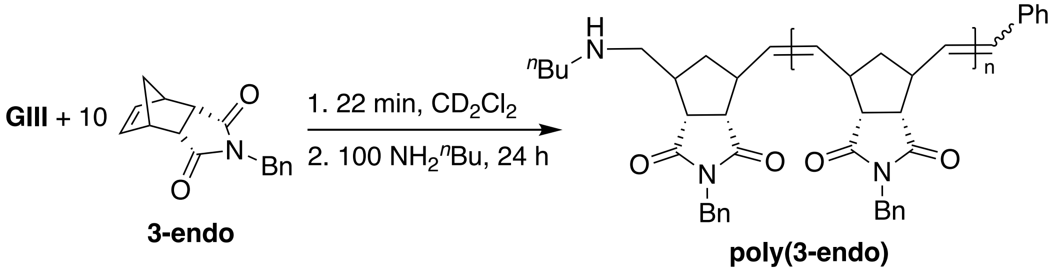

(a) Initial ratio of starting **GIII** to DMT (internal standard)

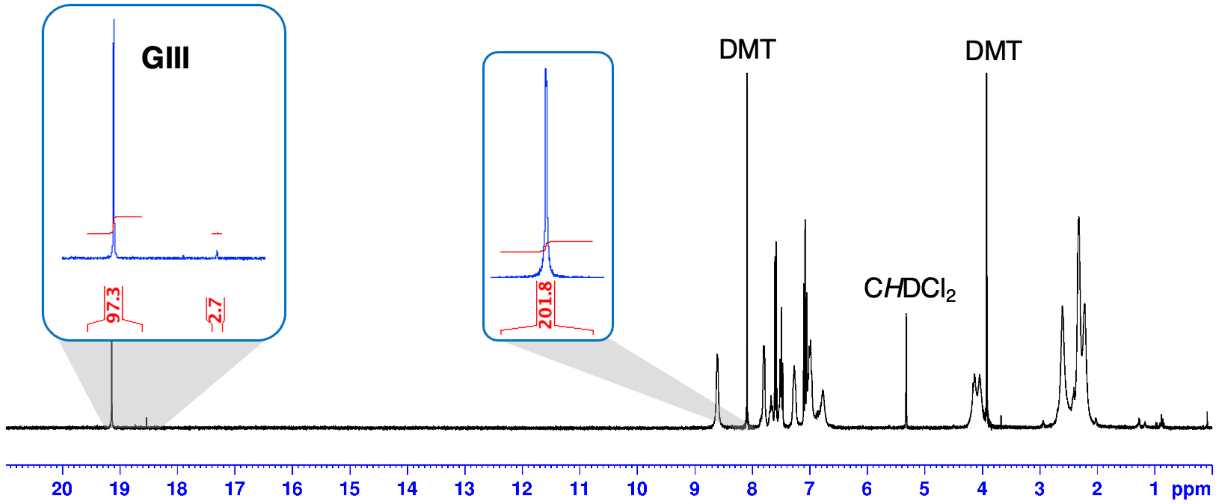

(b) t = 20 min during polymerization of **3-endo**.

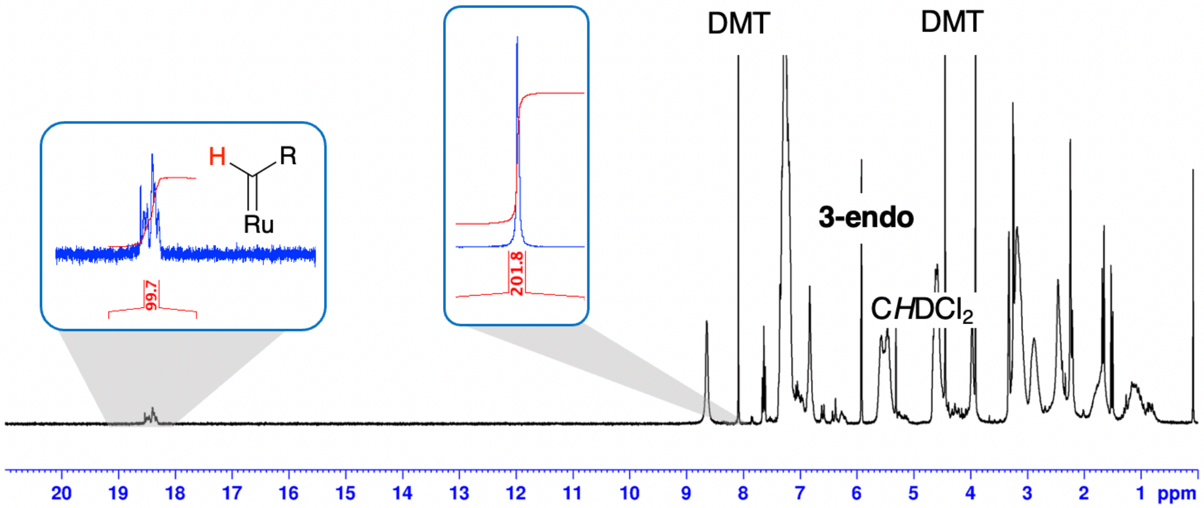

(Continues next page)

(c) t = 25 min (3 min after $NH_2{}^nBu$ addition)

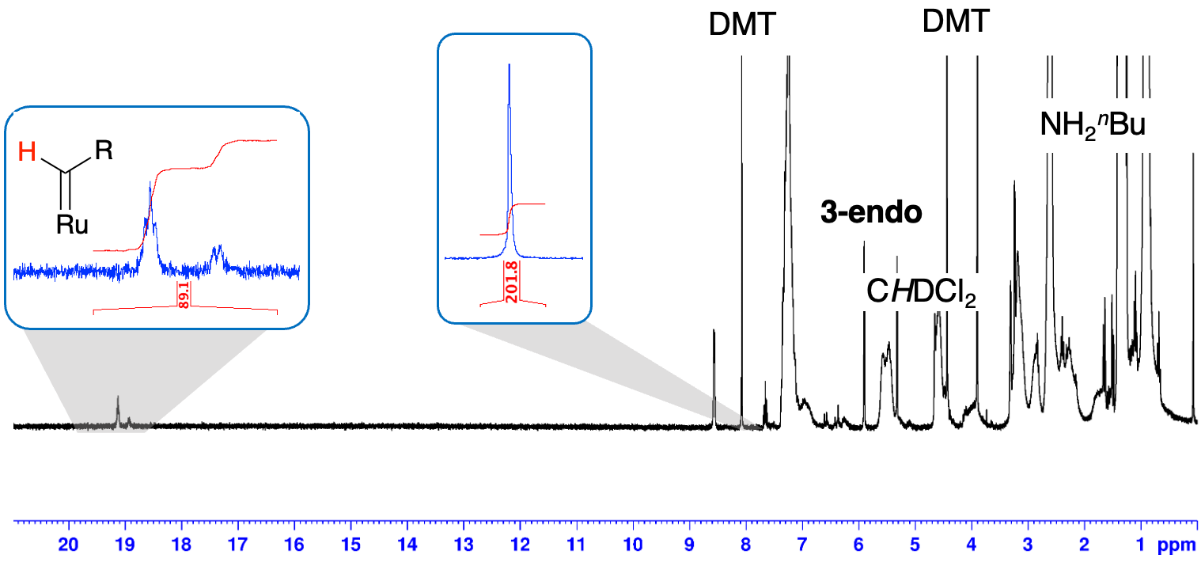

(d) t = 37 min (15 min after $NH_2{}^nBu$ addition)

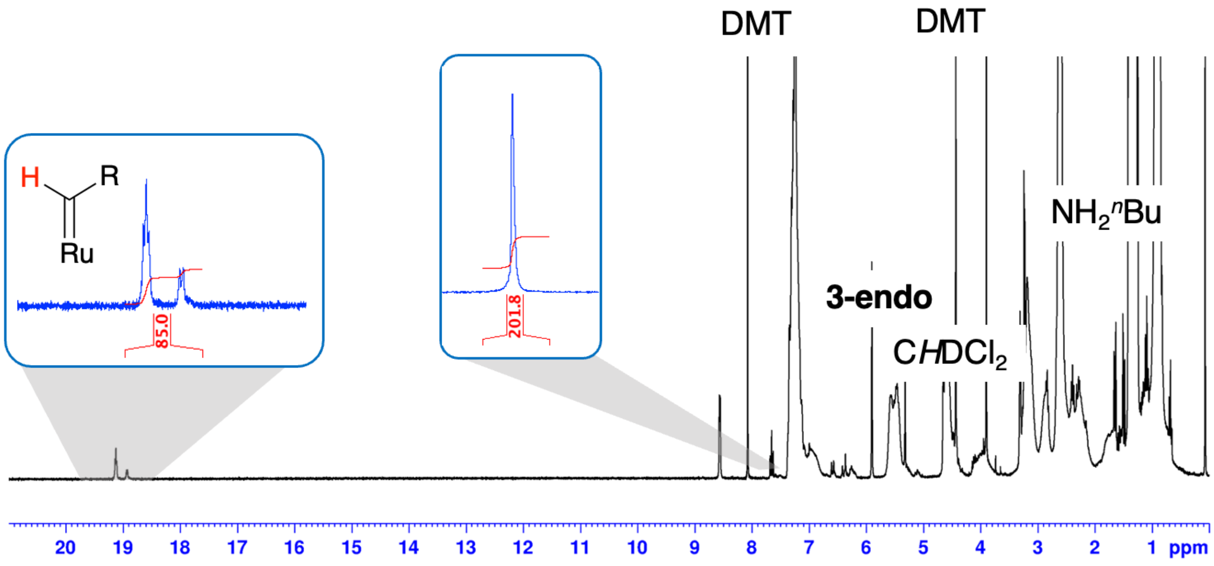

(Continues next page)

(e) t = 52 min (30 min after $NH_2{}^nBu$ addition)

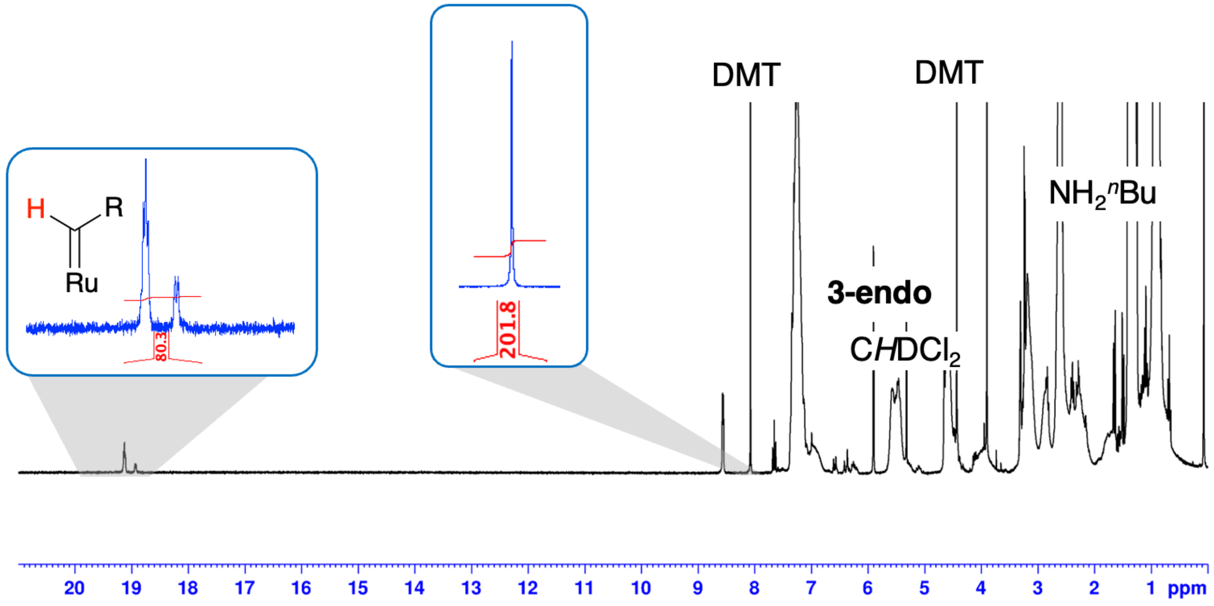

(f) t = 82 min (60 min after $NH_2{}^nBu$ addition)

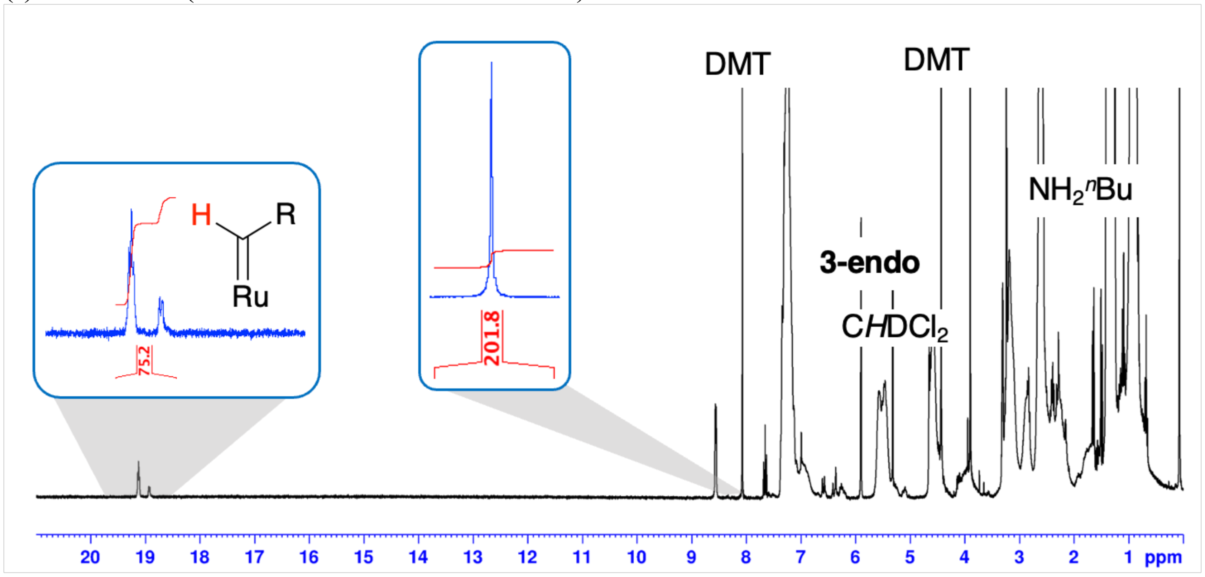

(Continues next page)

(g) t = 24 h after $NH_2{}^nBu$ addition

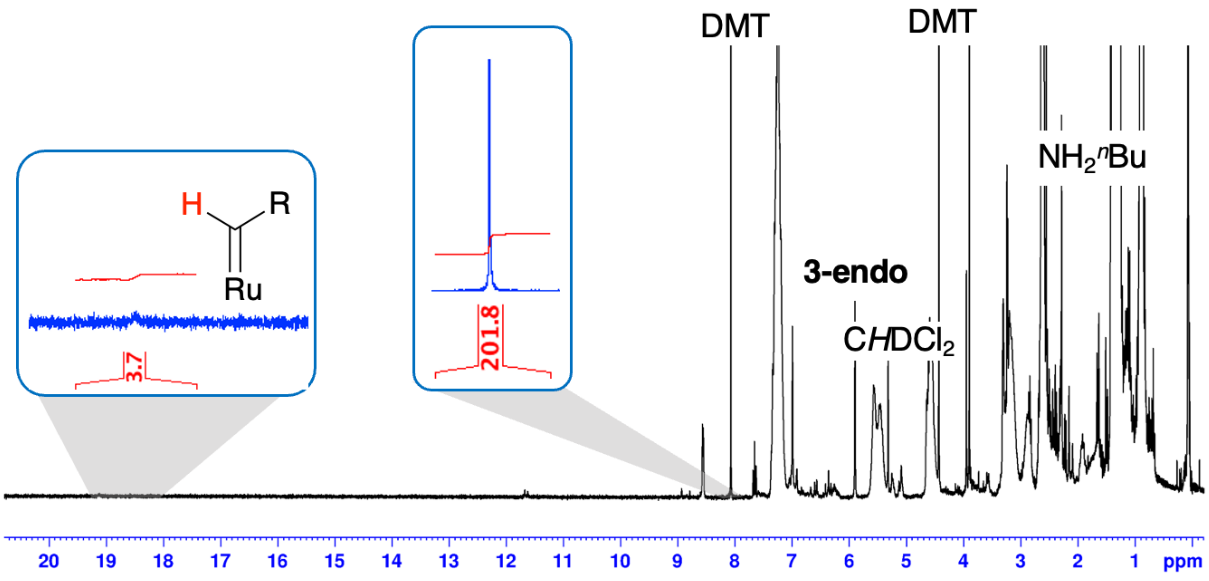

Figure S7. Quenching of a growing oligomer of **3-endo** with $NH_2{}^nBu$, showing decrease in [Ru]=*CHR* signal over time. (a) ^1H NMR spectrum (300 MHz, CD_2Cl_2) prior to adding **3-endo**. (b) Spectrum 20 min later. (c) Spectrum 3 min after adding $NH_2{}^nBu$. (d) 15 min after amine addition. (e) 30 min after amine addition. (f) 1 h after amine addition. (g) 24 h after amine addition.

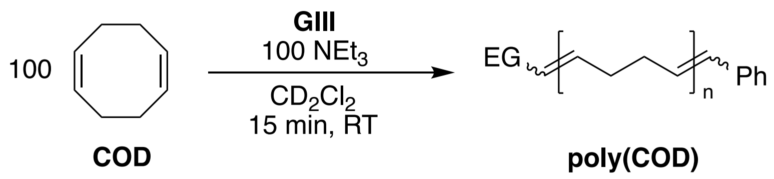

(a) Ratio of COD to anthracene (internal standard) at t = 0 h

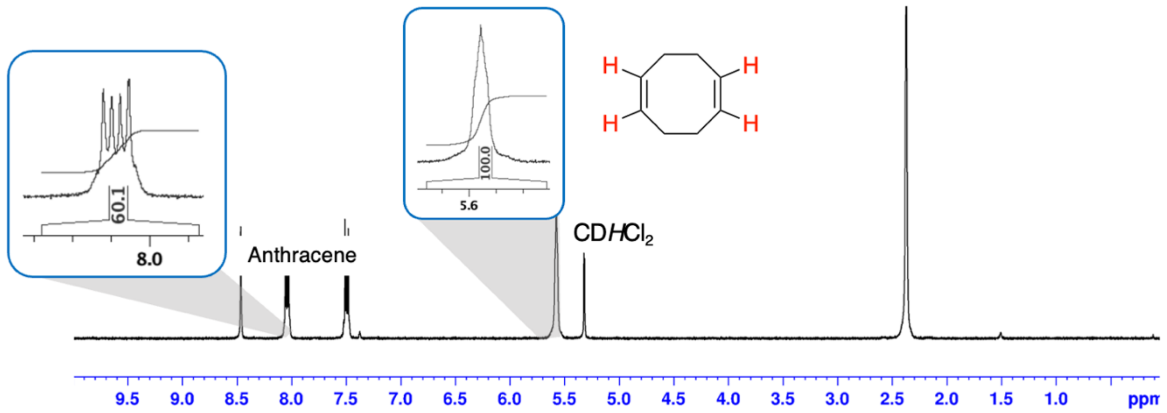

(b) t = 15 min

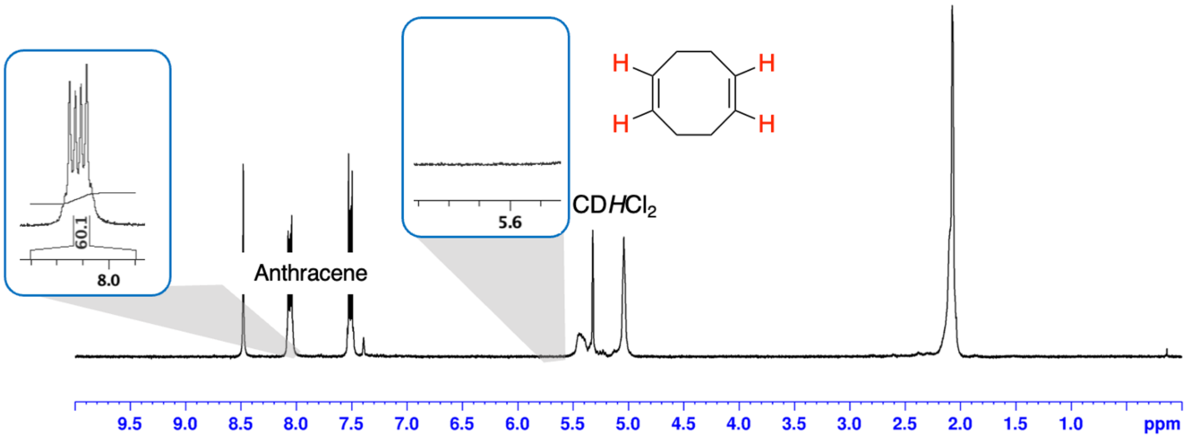

Figure S8. Representative quantitation of conversions in ROMP of **COD** with NEt_3 present. (a) ^1H NMR spectrum (300 MHz, CD_2Cl_2) prior to adding initiator **GIII**. (b) Spectrum 15 min after adding **GIII**, showing the absence of signals for the monomer.

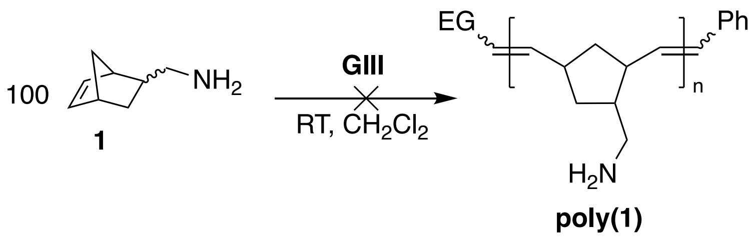

(a) Initial ratio of monomer **1** to anthracene (internal standard): t = 0 h

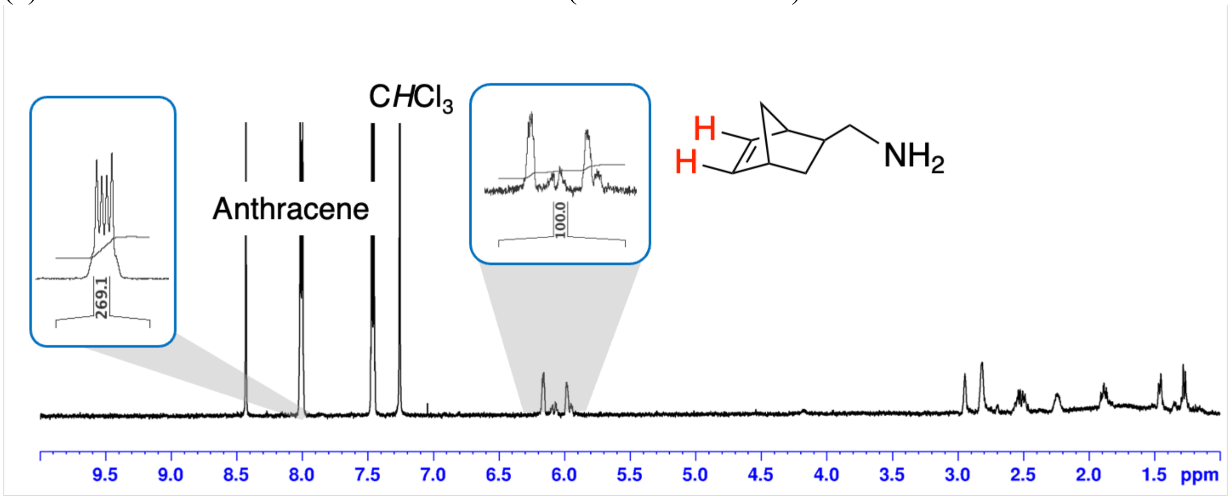

(b) t = 24 h

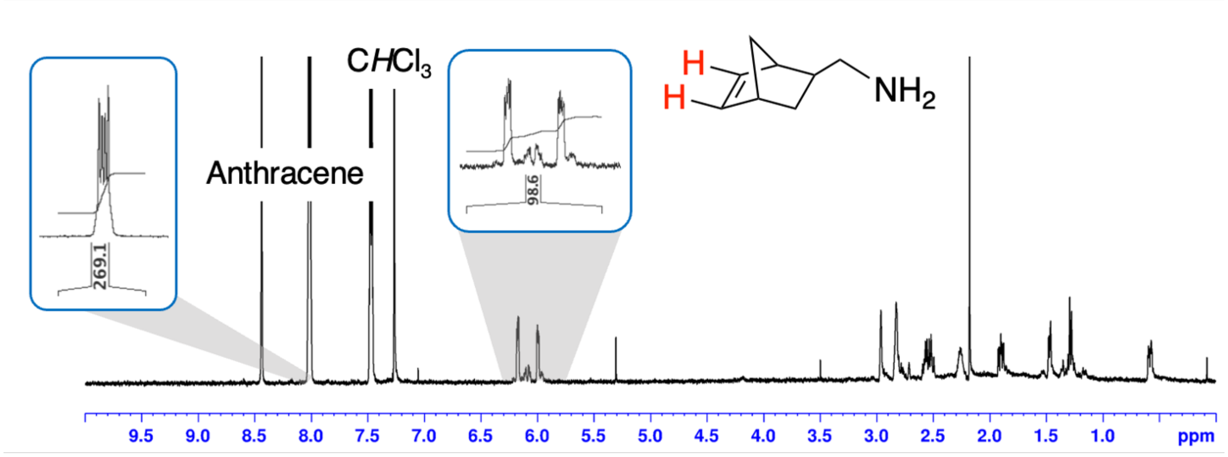

Figure S9. Representative quantitation of conversions in ROMP of **1**. (a) ^{1}H NMR spectrum (^{1}H NMR 500 MHz, $CDCl_3$) prior to adding initiator **GIII**. (b) Spectrum 24 h later, showing the remaining signals for the monomer.

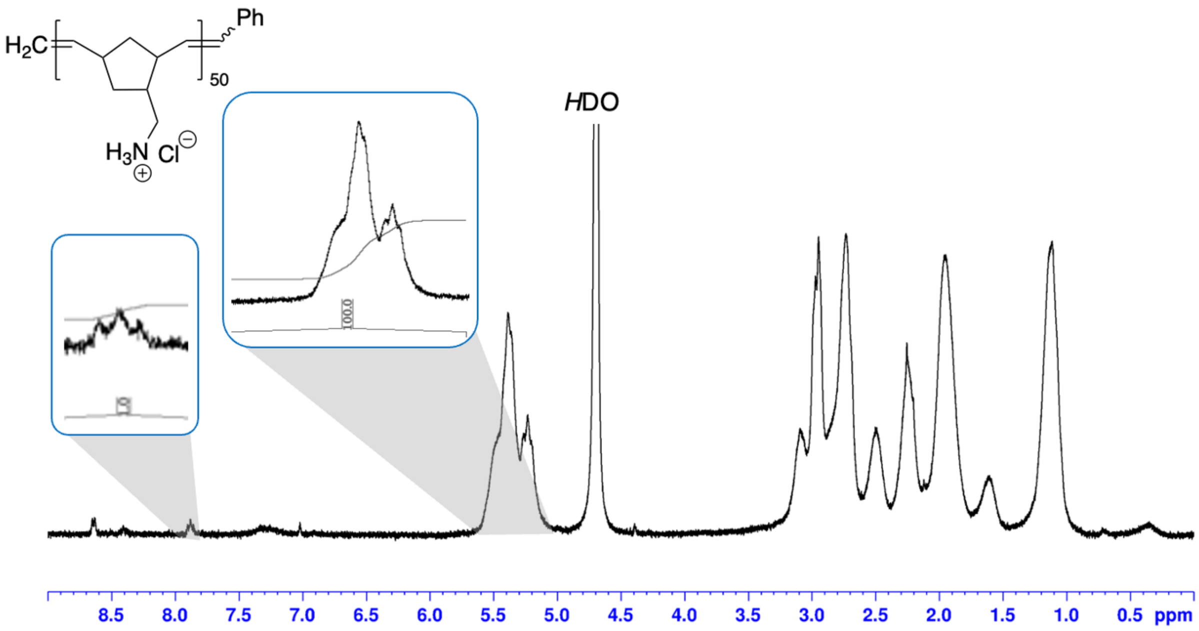

Figure S10. ^{1}H NMR spectrum of **poly(1)•HCl** (300 MHz, D_2O).

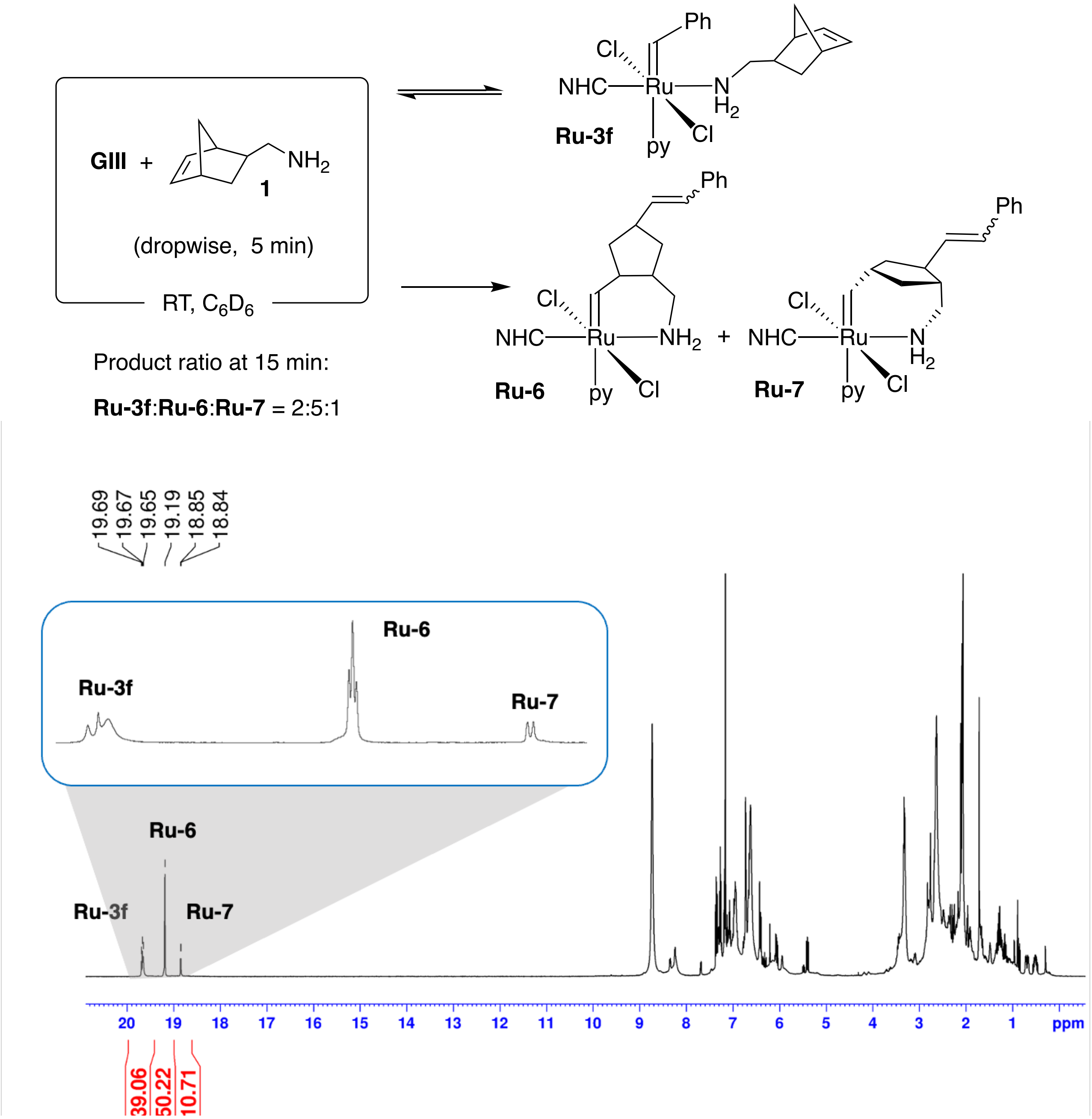

Figure S11. ¹H NMR spectrum (500 MHz, C_6D_6) showing in situ-generated **Ru-6**, **Ru-7**, and **Ru-3f**.

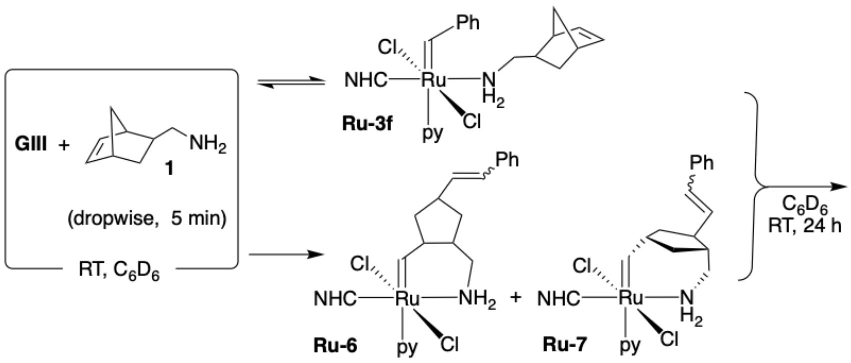

	Ru-3f (%)	Ru-6 (%)	Ru-7 (%)
t = 0 h	37	52	11
t = 24 h	28	38	9
Δ	9	14	2

(a) Initial proportions of Ru species relative to DMT (internal standard).

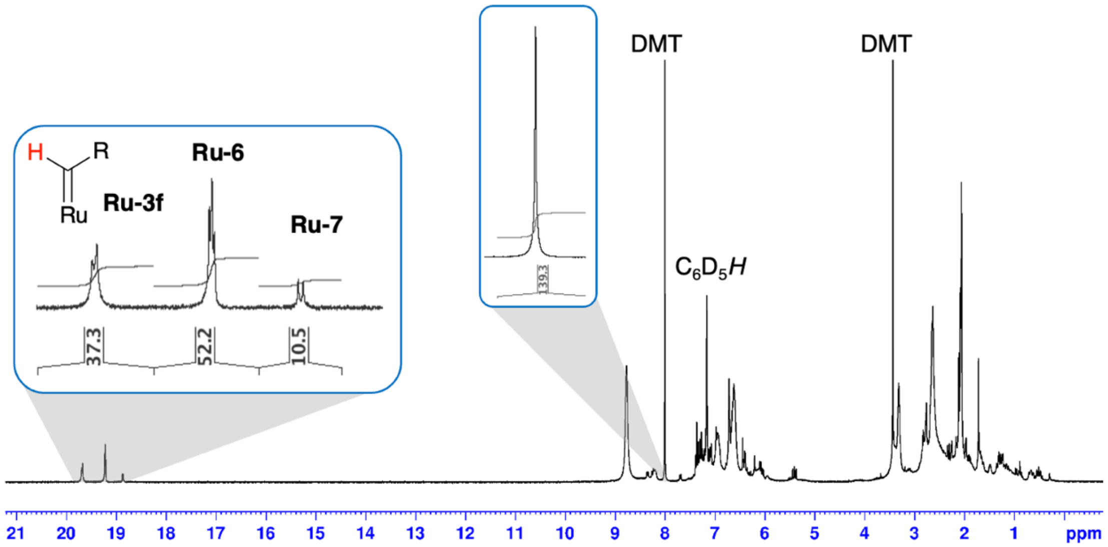

(b) Proportion of Ru species at 24 h.

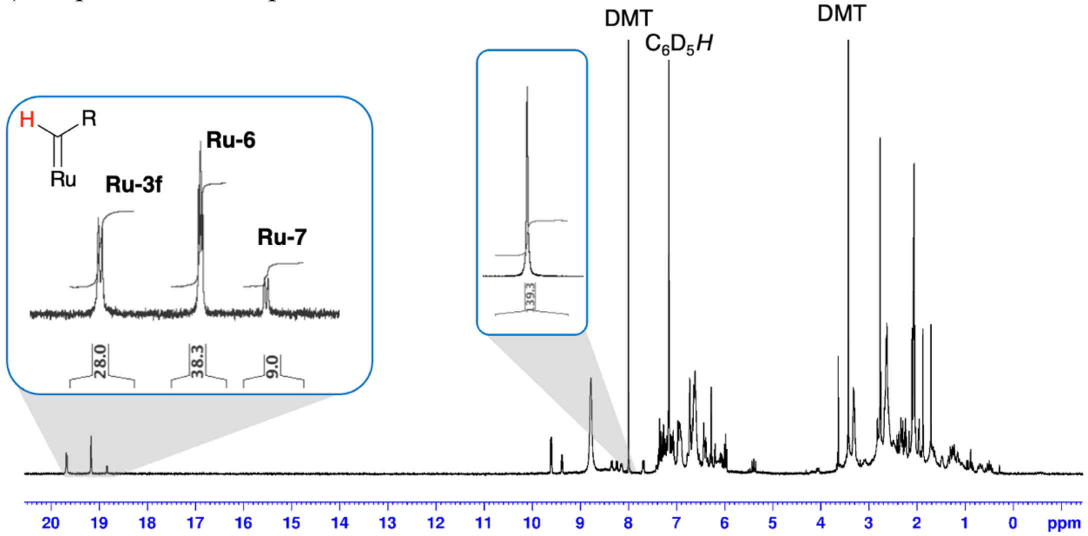

Figure S12. ^{1}H NMR spectra of norbornene-methylamine complexes: adduct **Ru-3f** and chelates **Ru-6/7** (300 MHz, C_6D_6). (a) Immediately after adding **1** to **GIII**. (b) After mixing for 24 h.

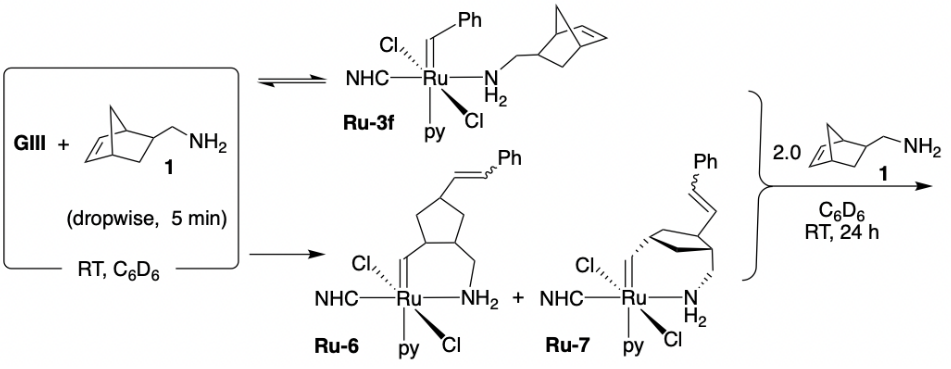

	Ru-3f (%)	Ru-6 (%)	Ru-7 (%)
t = 0 h	25	63	12
t = 24 h	0	8	5
Δ	25	55	7

(a) Initial ratio of Ru species to DMT (internal standard).

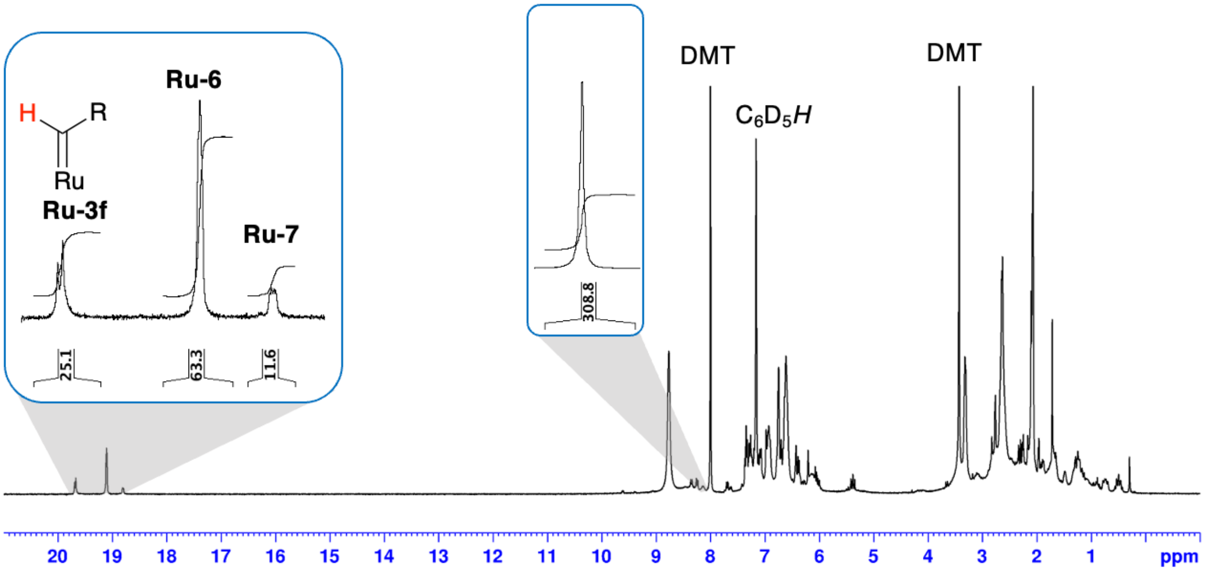

(b) t = 24 h

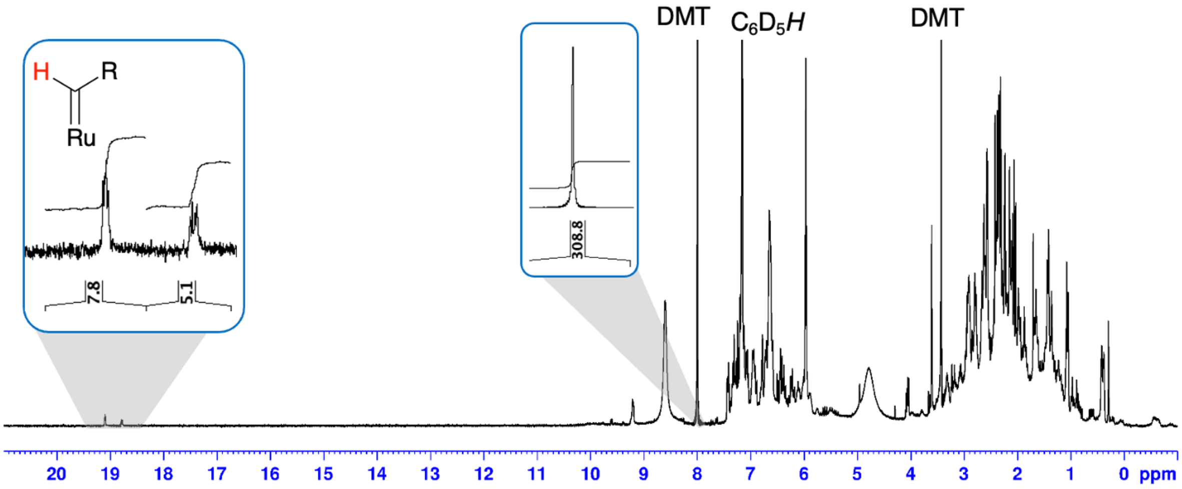

Figure S13. Reaction of Ru methylamine norbornene compounds **Ru-6, Ru-7** and **Ru-3f** with 1 equiv **1**. ^{1}H NMR spectra (300 MHz, C_6D_6): (a) Immediately after generating the mixture. (b) After adding **1** and mixing for 24 h.

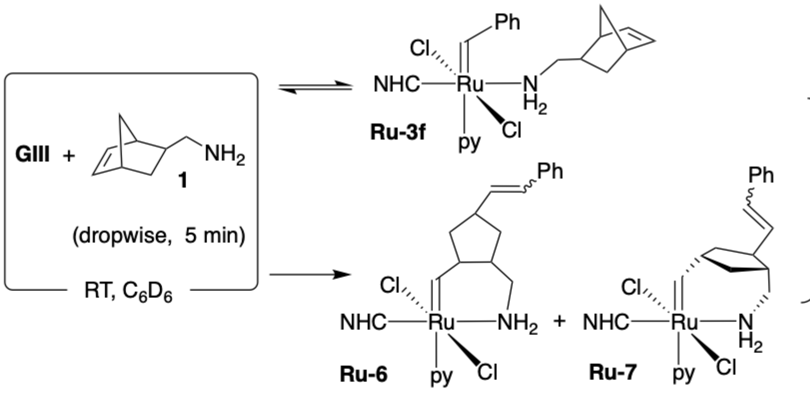

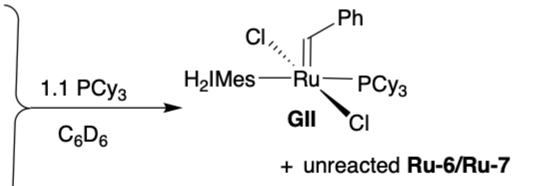

	**GII** (%)	**Ru-3f** (%)	**Ru-6** (%)	**Ru-7** (%)
t = 0 min	0	36	53	12
t = 5 min	25	0	41	9
t = 1 h	30	0	44	7
t = 24 h	24	0	23	8
Δ	+ 24	36	30	4

(a) Initial ratio of Ru species to TMB (internal standard).

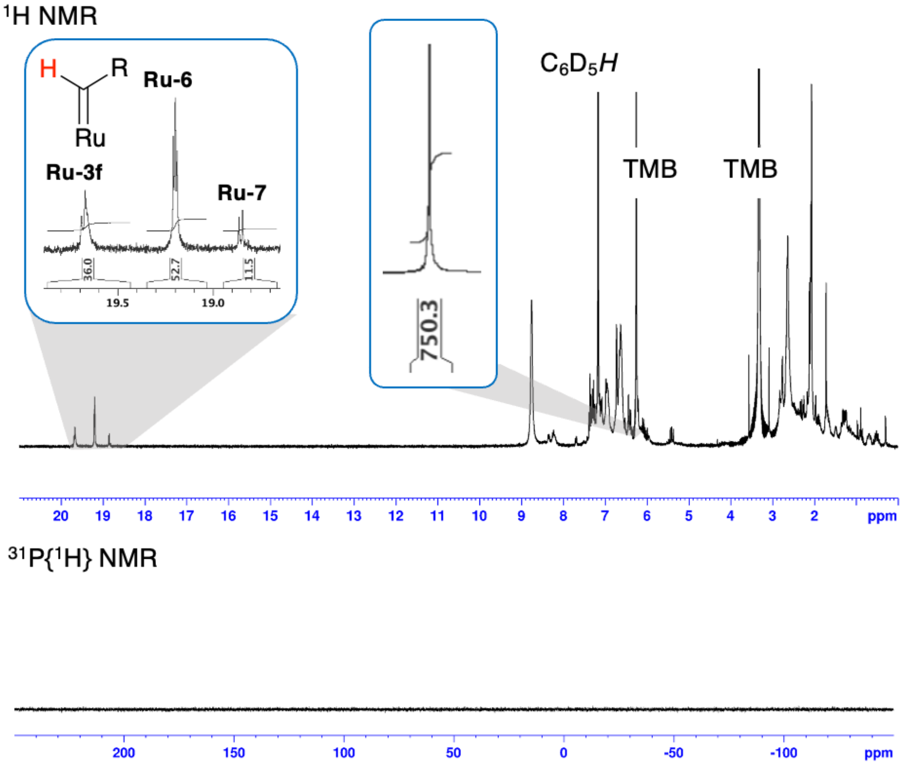

(Continues on next page)

(b) t = 5 min

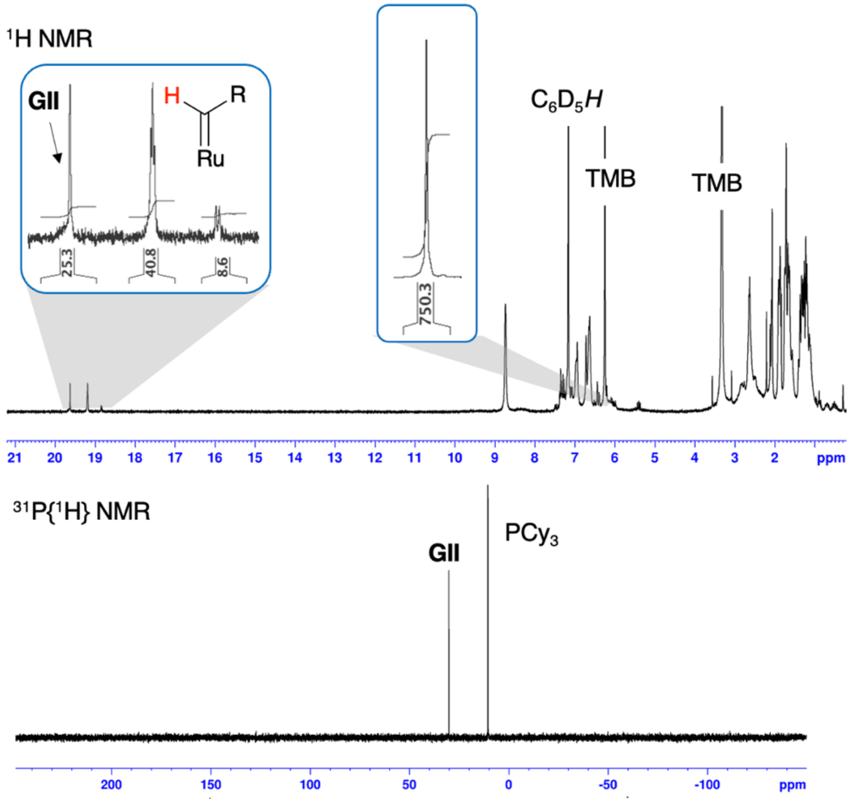

(c) t = 1 h

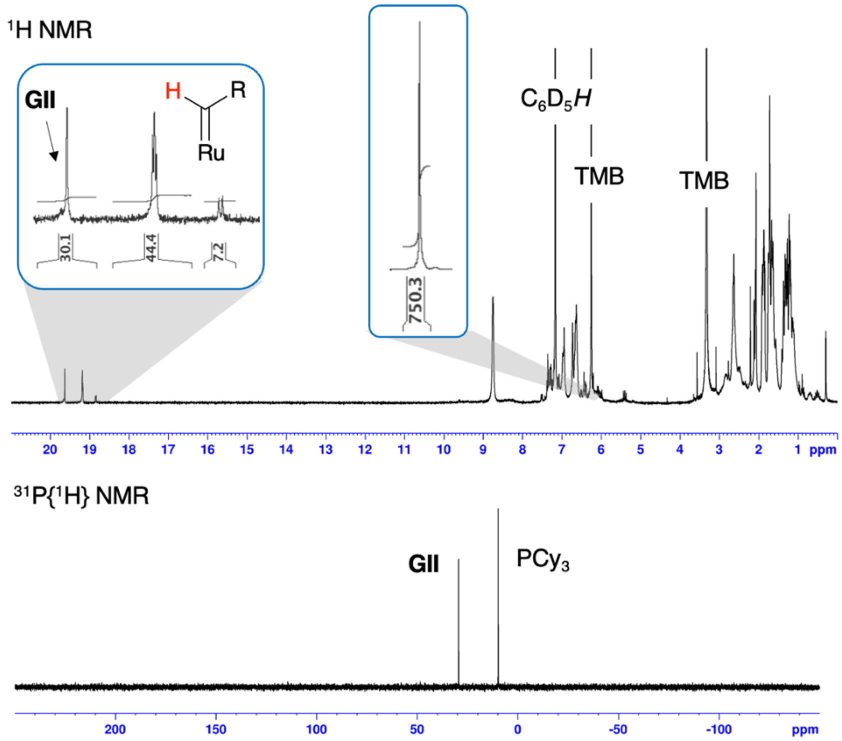

(Continues on next page)

(d) t = 24 h

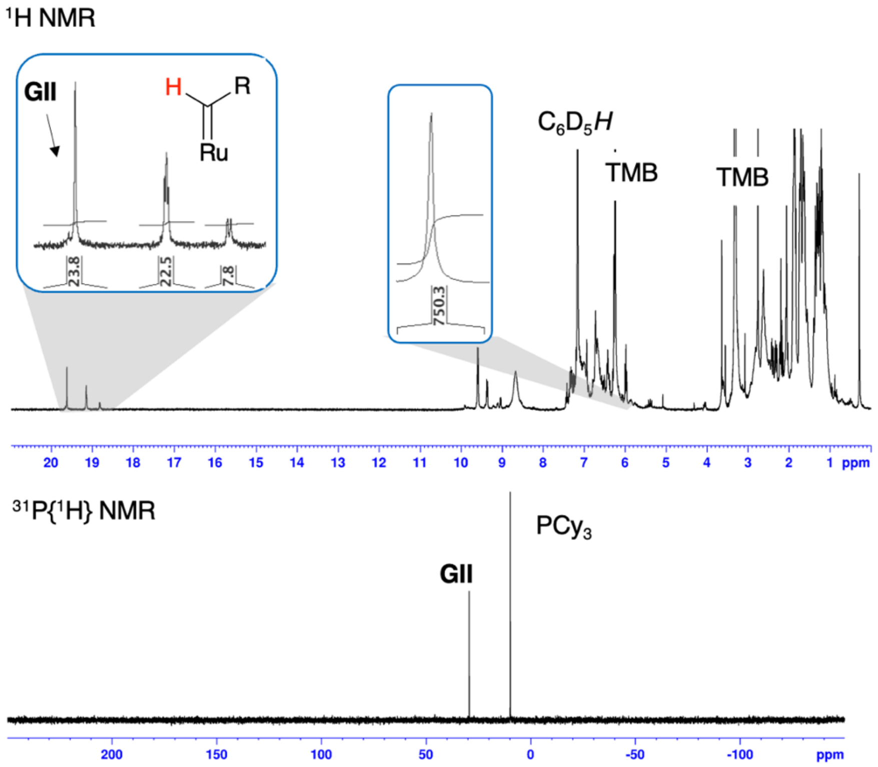

Figure S14. ^{1}H and $^{31}P\{^{1}H\}$ NMR spectra (300 or 121 MHz; C_6D_6) for the reactions of **Ru-6, Ru-7,** and **Ru-3f** with PCy_3. (a) Prior to adding PCy_3. (b) 5 min after addition. (c) 1 h after adding phosphine. (d) 24 h after addition.

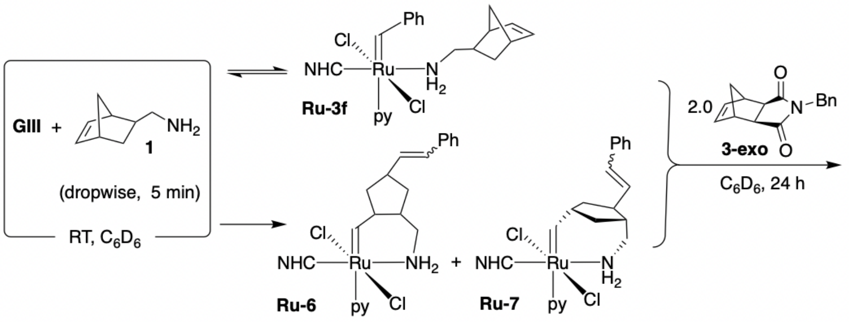

	Ru-3f (%)	Ru-6 (%)	Ru-7 (%)
t = 0 h	38	51	11
t = 24 h	0	41	8
Δ	38	10	3

(a) Ratio of Ru species to DMT (internal standard).

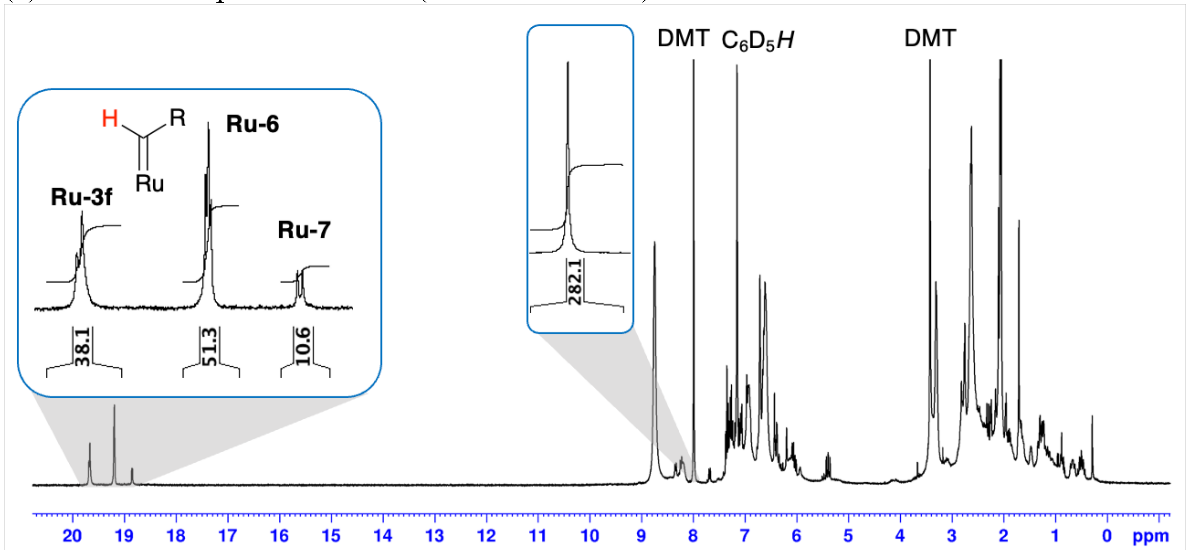

(b) t = 24 h

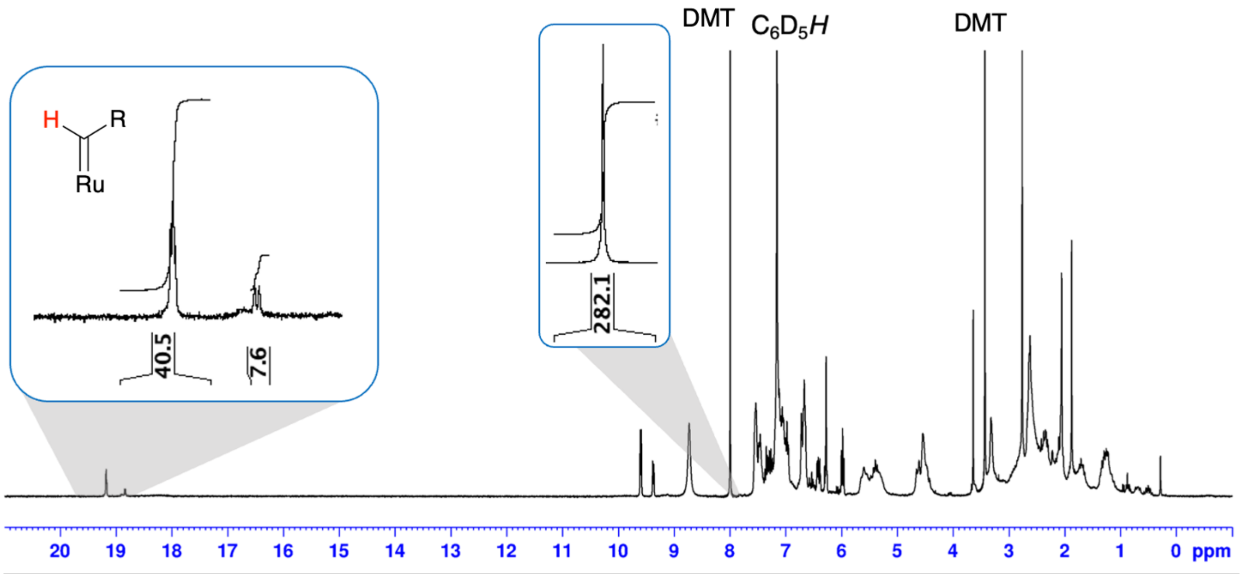

Figure S15. ^{1}H NMR spectra (300 MHz, C_6D_6) for the reaction of **Ru-6, Ru-7,** and **Ru-3f** with **3-exo**. (a) Prior to adding monomer. (b) After mixing for 24 h.

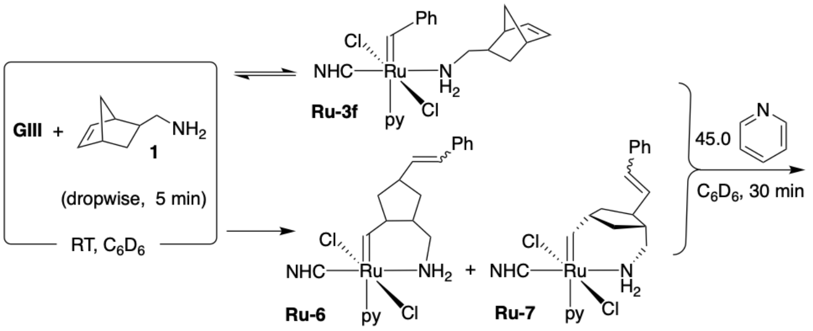

	Ru-3f (%)	Ru-6 (%)	Ru-7 (%)
t = 0 min	43	48	9
t = 30 min	26	50	10
Δ	17	+2	+1

(a) Ratio of Ru species to DMT (internal standard).

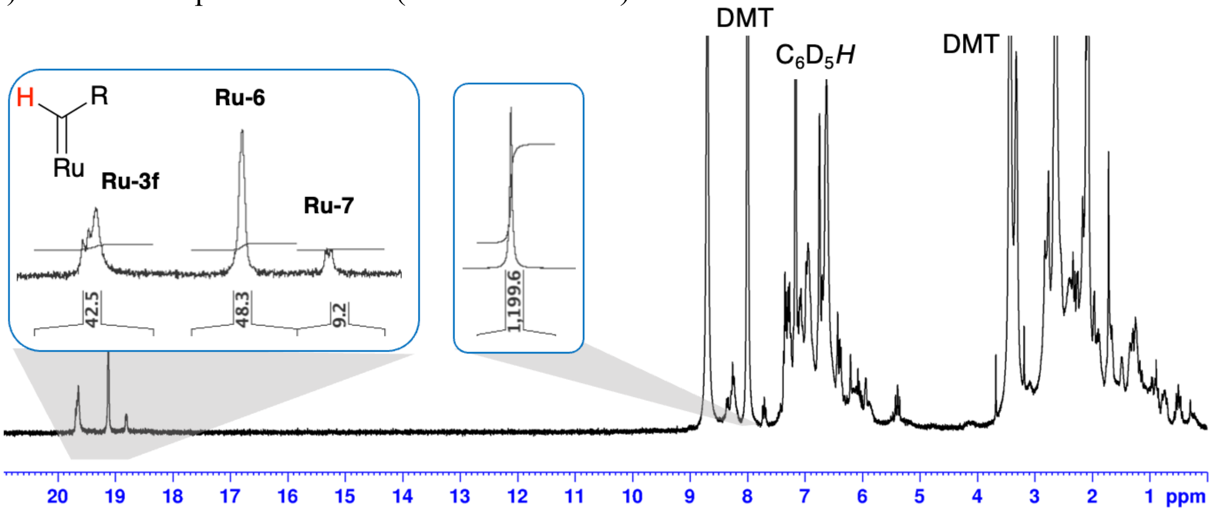

(b) t = 30 min

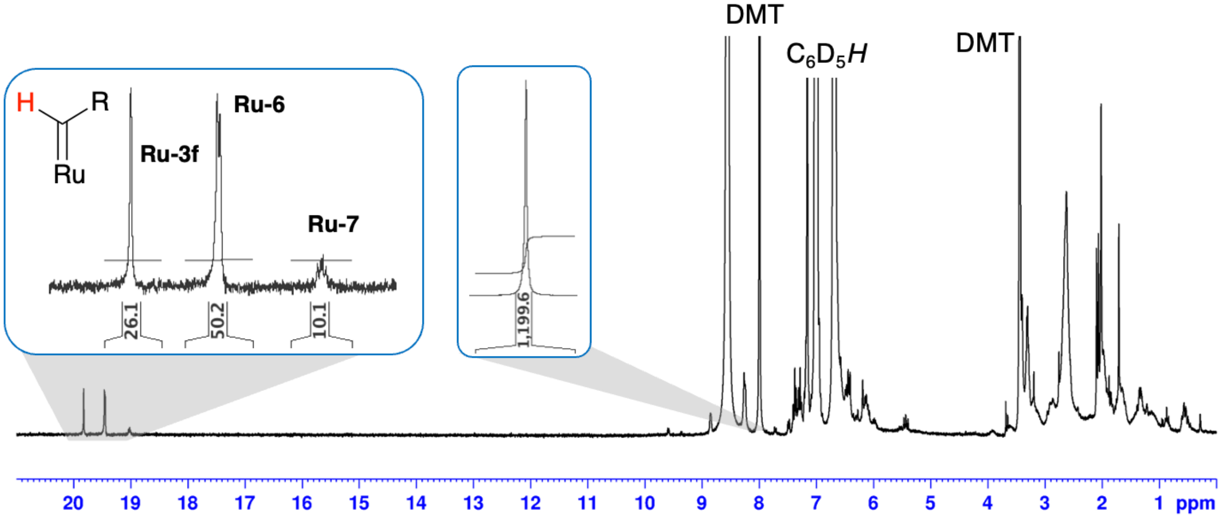

Figure S16. ^{1}H NMR spectra (300 MHz, C_6D_6) for the reaction of **Ru-6, Ru-7,** and **Ru-3f** with pyridine. (a) Prior to adding pyridine (b) After mixing for 30 min.

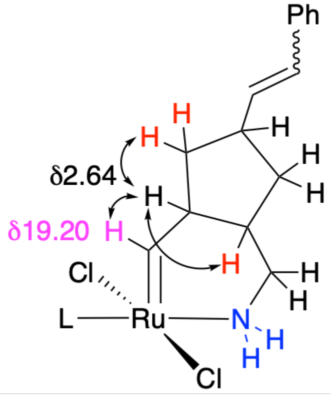

^{1}H-^{1}H COSY	^{1}H-^{13}C HSQC	DEPT 135
(δ19.20, δ2.64)	(δ2.64, δ40.4)	δ40.4 up (CH)
(δ2.64, δ1.65)	(δ1.65, δ19.3)	δ19.3 up (CH)
(δ2.64, δ0.53)	(δ0.53, δ37.5)	δ37.5 down (CH_2)

(a) ^{1}H-^{1}H COSY 45 highlighting cross-peaks of **Ru-6** (L = H_2IMes).

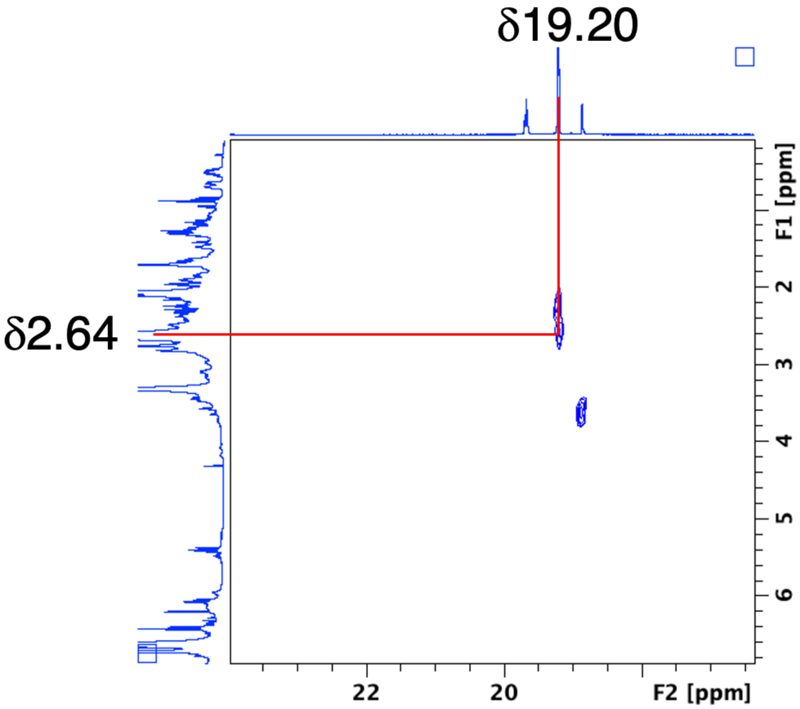

(Continues on next page)

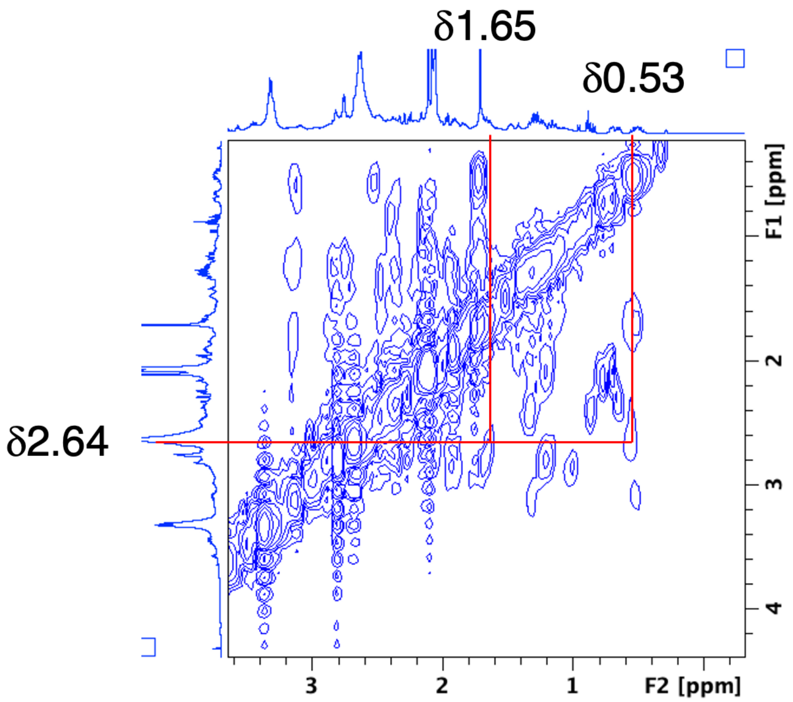

(b) ^{1}H-^{13}C HSQC spectrum showing cross-peaks of **Ru-6** (^{13}C axis is 135 DEPT spectrum).

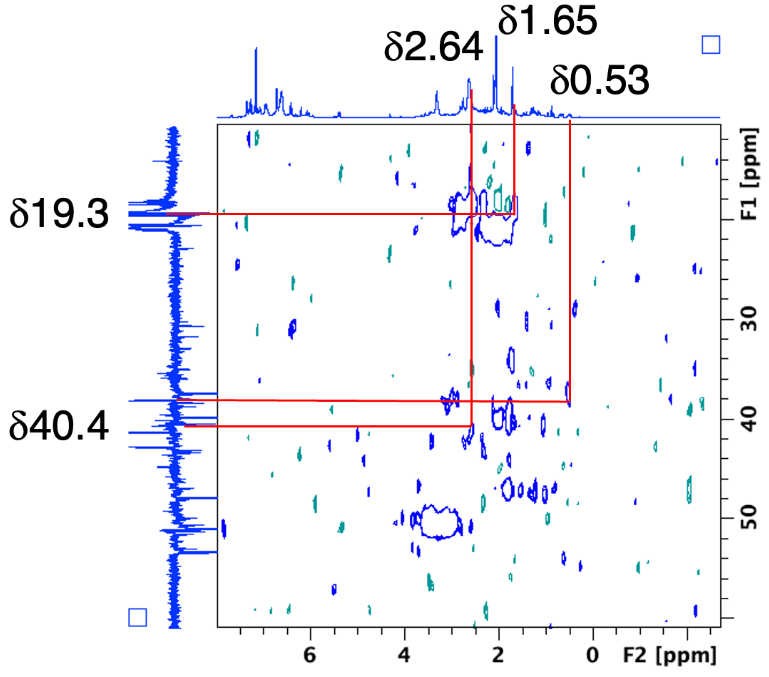

Figure S17. 2D NMR spectra supporting structure of Ru-amine chelate **Ru-6** (500 MHz, C_6D_6). (a) ^{1}H-^{1}H COSY 45. (b) ^{1}H-^{13}C HSQC.

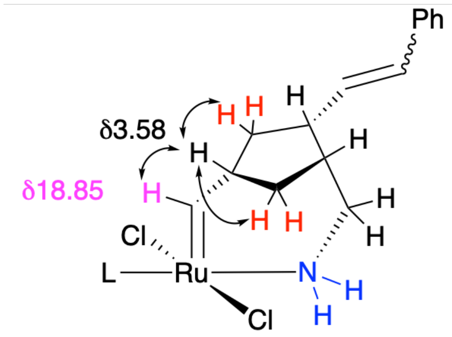

^{1}H-^{1}H COSY	^{1}H-^{13}C HSQC	DEPT 135
(δ18.85, δ3.58)	(δ3.58, δ71.5)	δ71.5 up (CH)
(δ3.58, δ1.10)	(δ1.32, δ47.7)	δ47.7 down (CH_2)
(δ3.58, δ1.32)	(δ1.10, δ47.9)	δ47.9 down (CH_2)

(a) ^{1}H-^{1}H COSY 45 highlighting cross-peaks of **Ru-7** (L = H_2IMes).

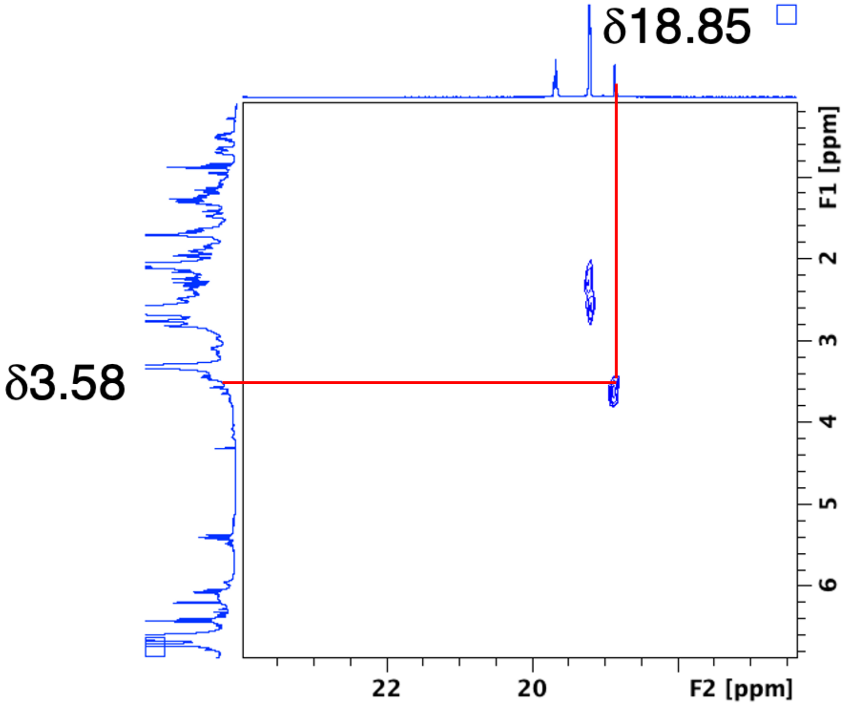

(Continues on next page)

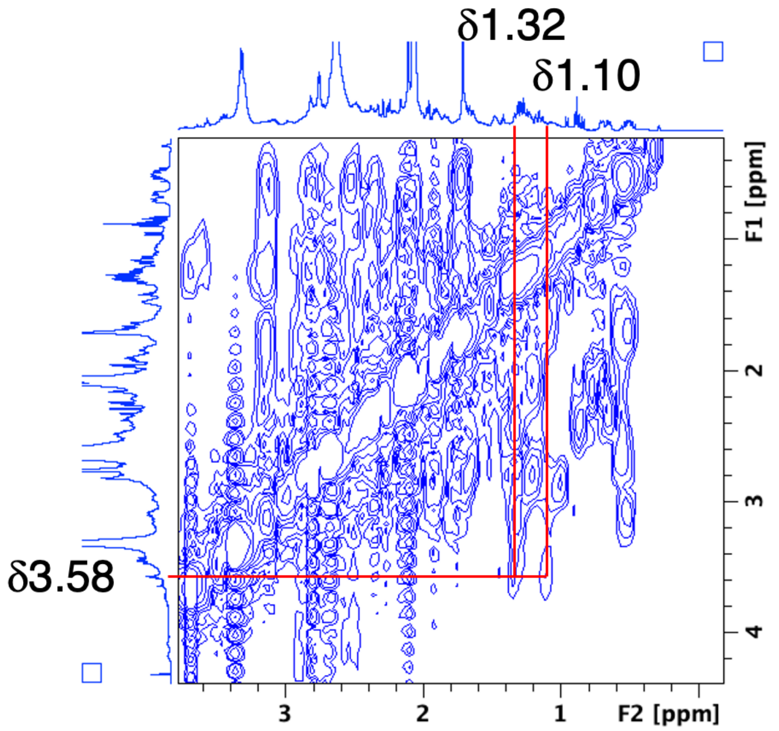

(b) ^{1}H-^{13}C HSQC highlighting cross-peaks of **Ru-7** (^{13}C axis is 135 DEPT spectrum).

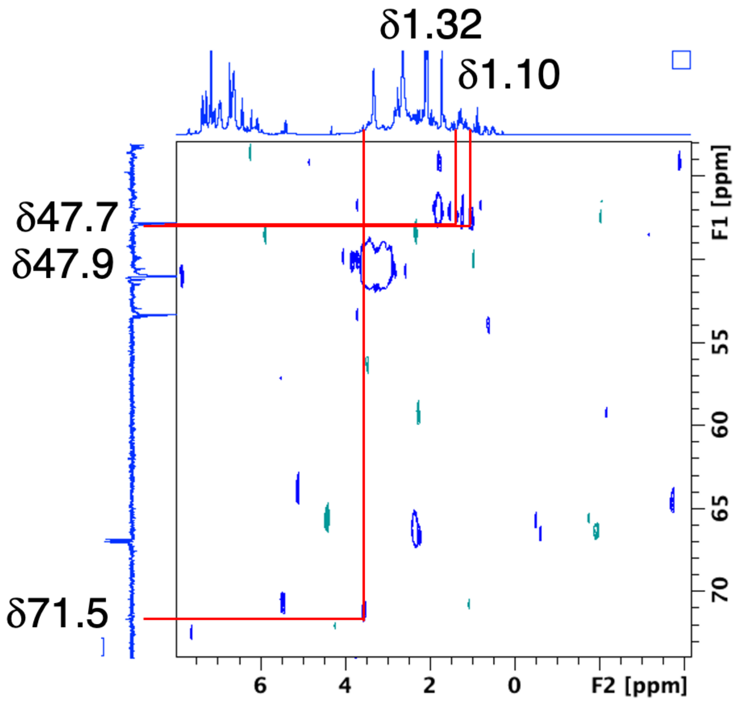

Figure S18. 2D NMR spectra supporting structure of Ru-amine chelate **Ru-7** (500 MHz, C_6D_6). (a) ^{1}H-^{1}H COSY 45. (b) ^{1}H-^{13}C HSQC.

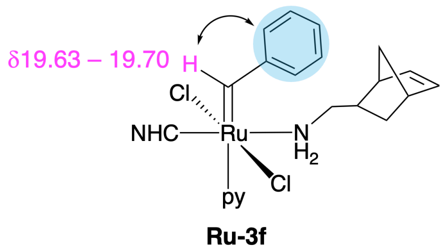

(a) ^{1}H-^{13}C HMBC highlighting cross-peaks of **Ru-3f** (^{13}C axis is 135 DEPT spectrum).

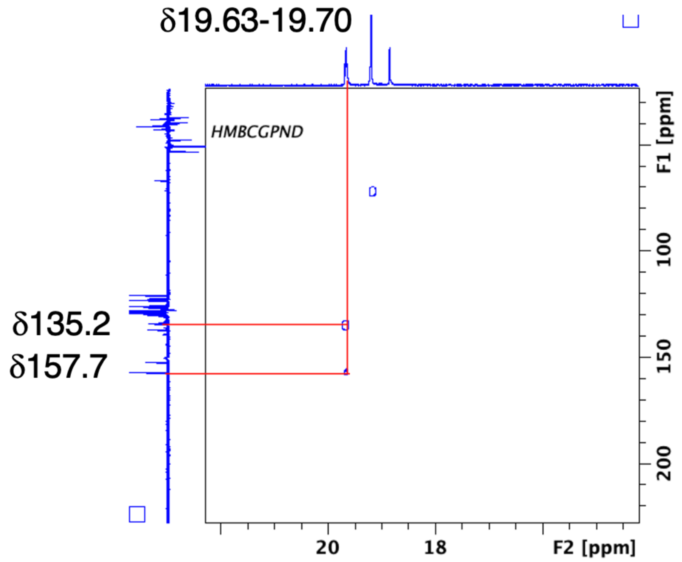

(b) ^{1}H NMR of Ru methylamine norbornene species (bottom) compared to **GIII**

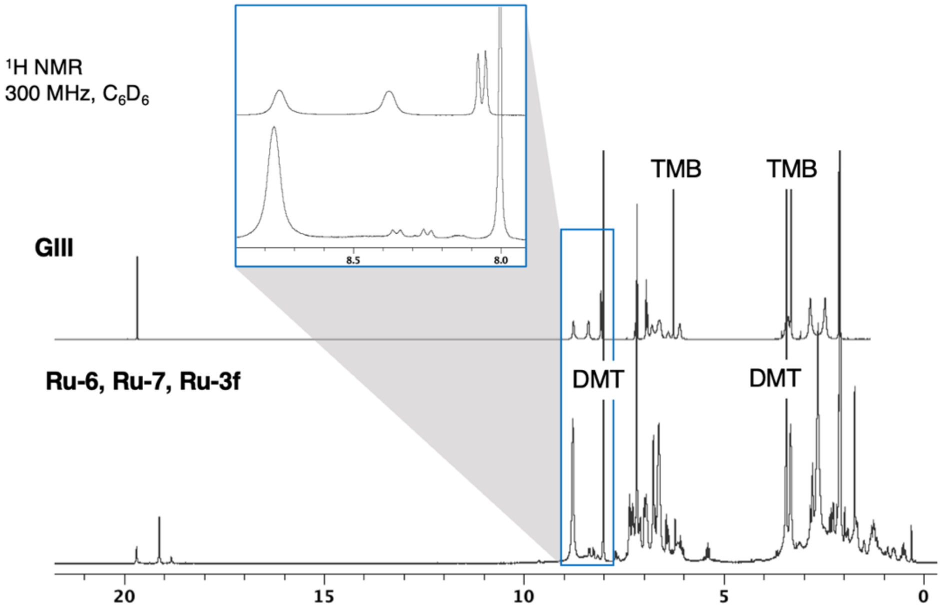

Figure S19. 2D NMR spectrum for Ru-amine adduct **Ru-3f** (300 MHz, C_6D_6). (a) ^{1}H-^{13}C HMBC showing correlations to aromatic region. (b) Evidence for absence of **GIII**.

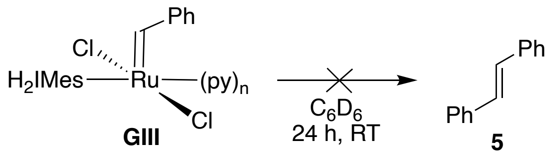

(a) Initial ratio of **GIII** to TMB (internal standard)

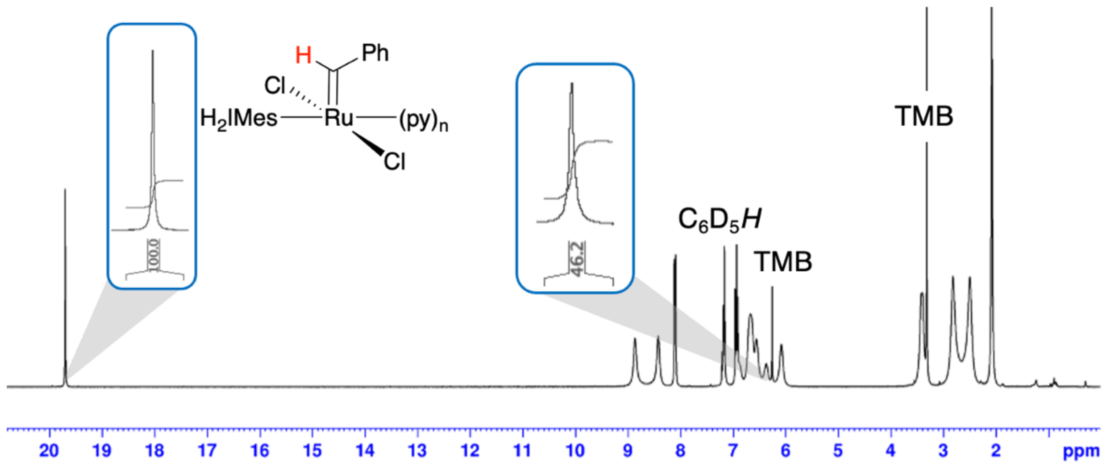

(b) t = 1 h

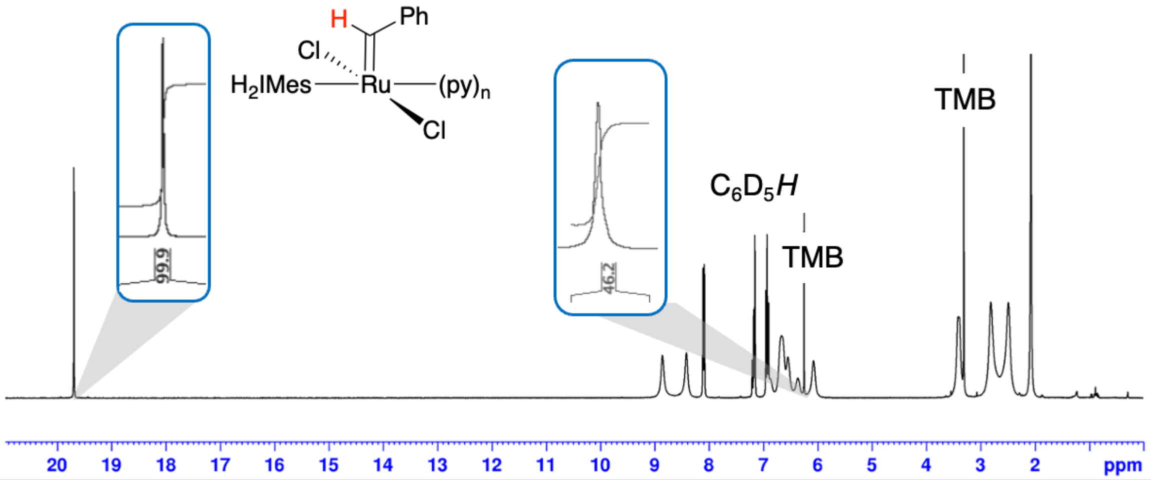

(Continues on next page)

(c) t = 24 h

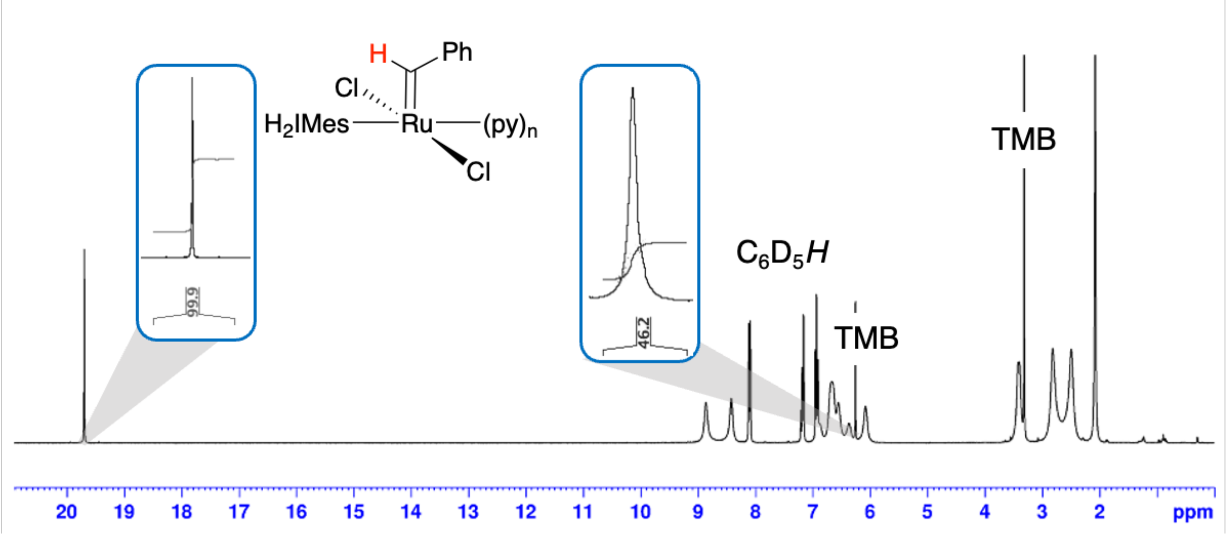

Figure S20. ^{1}H NMR spectrum of **GIII** (300 MHz, C_6D_6). (a) Before mixing. (b) After 1 h mixing. (c) After 24 h mixing.

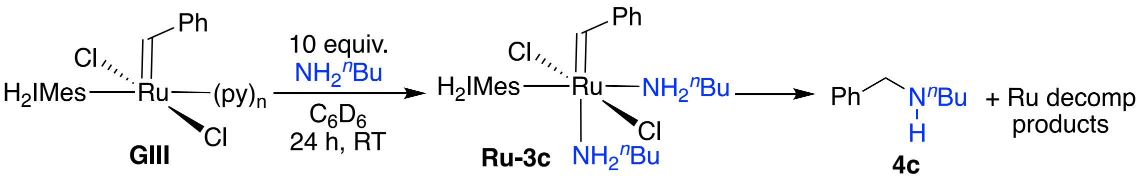

(a) Initial ratio of **GIII** to TMB as internal standard (prior to amine addition)

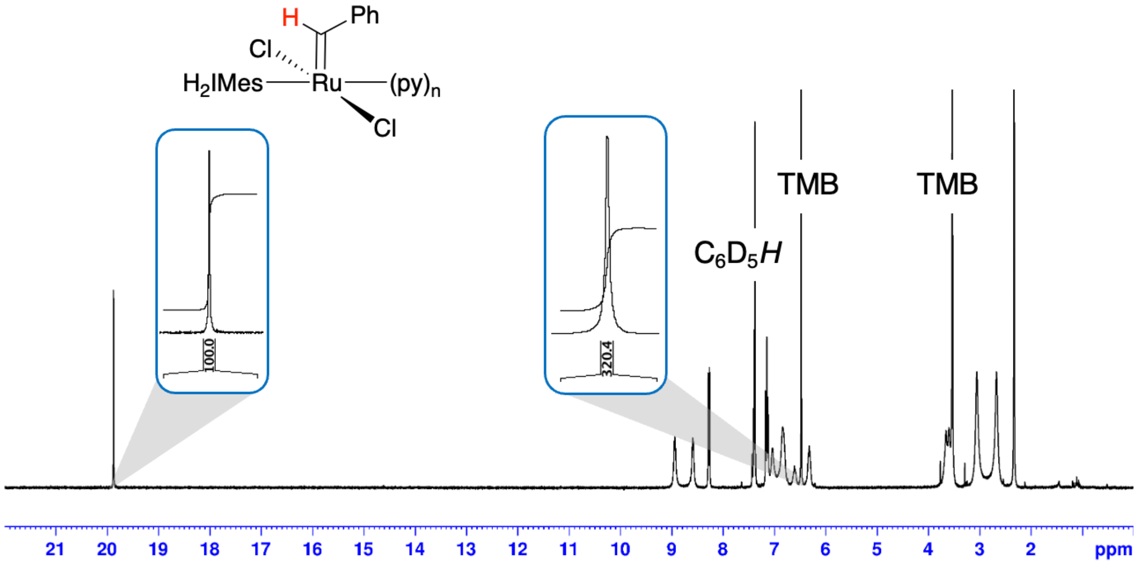

(b) t = 1 h

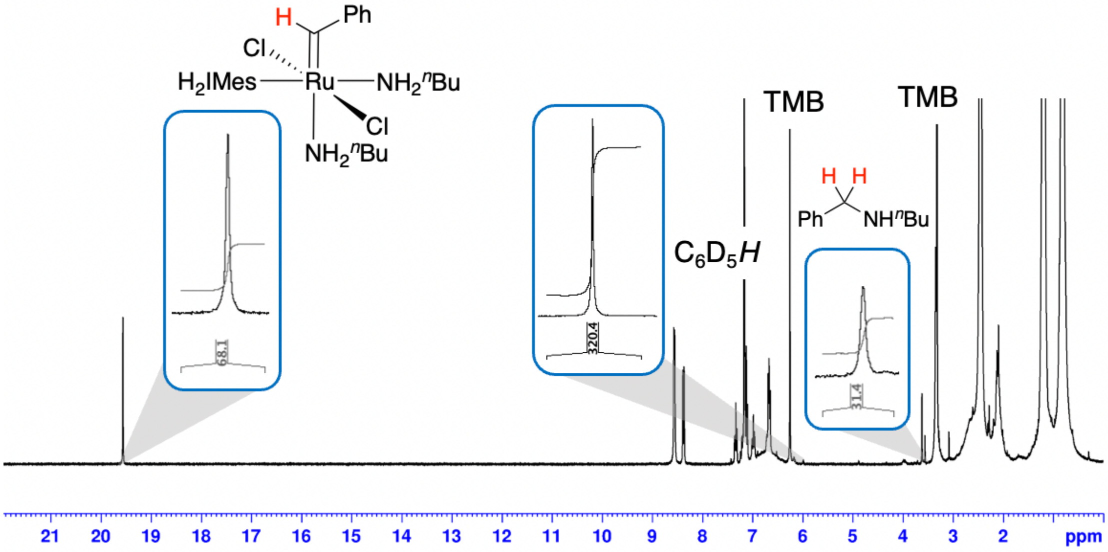

(Continues on next page)

(c) t = 24 h

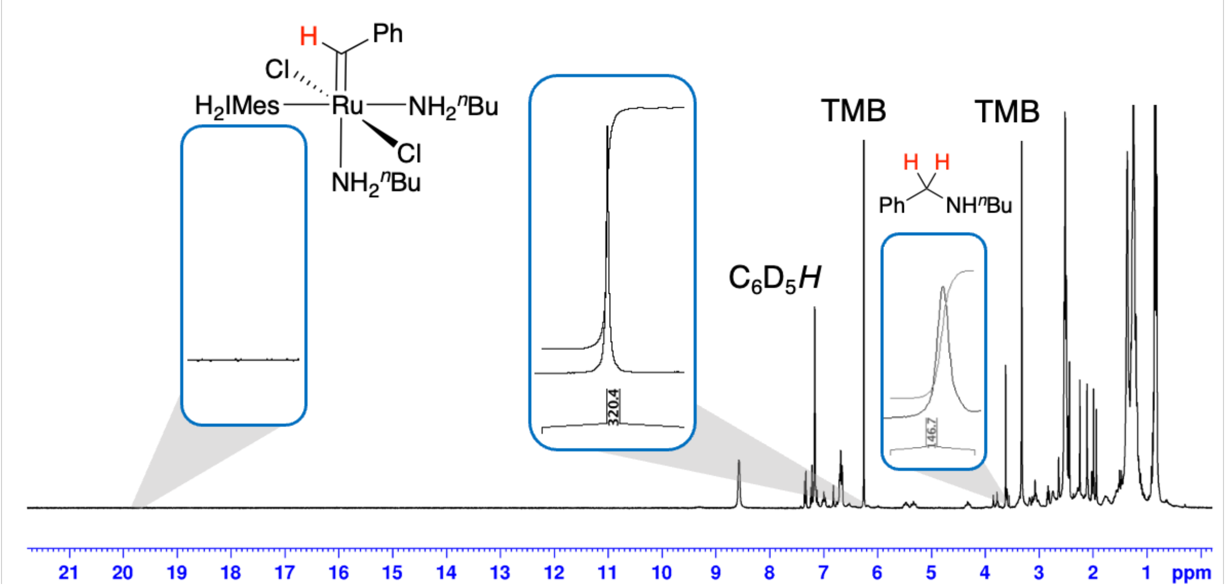

Figure S21. ^{1}H NMR spectra of the reaction of **GIII** with $NH_2{}^n$Bu. (300 MHz, C_6D_6). (a) Before addition of $NH_2{}^n$Bu. (b) After mixing with $NH_2{}^n$Bu for 1 h. (c) After 24 h.

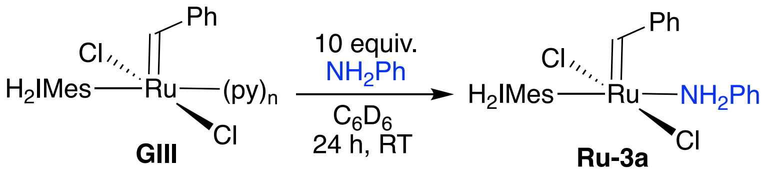

(a) Initial ratio of **GIII** to TMB as internal standard (prior to amine addition)

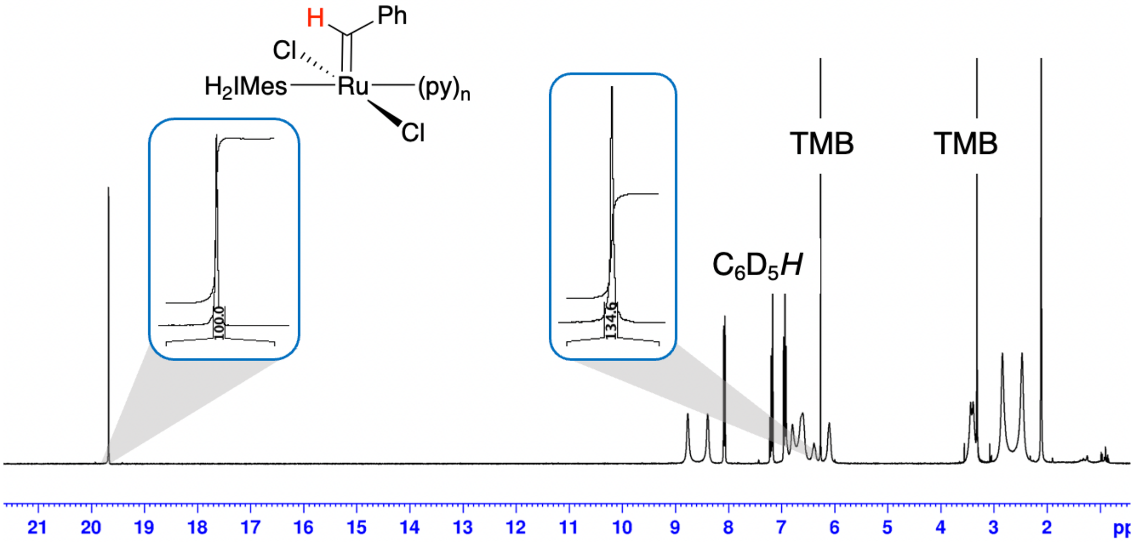

(b) t = 1 h

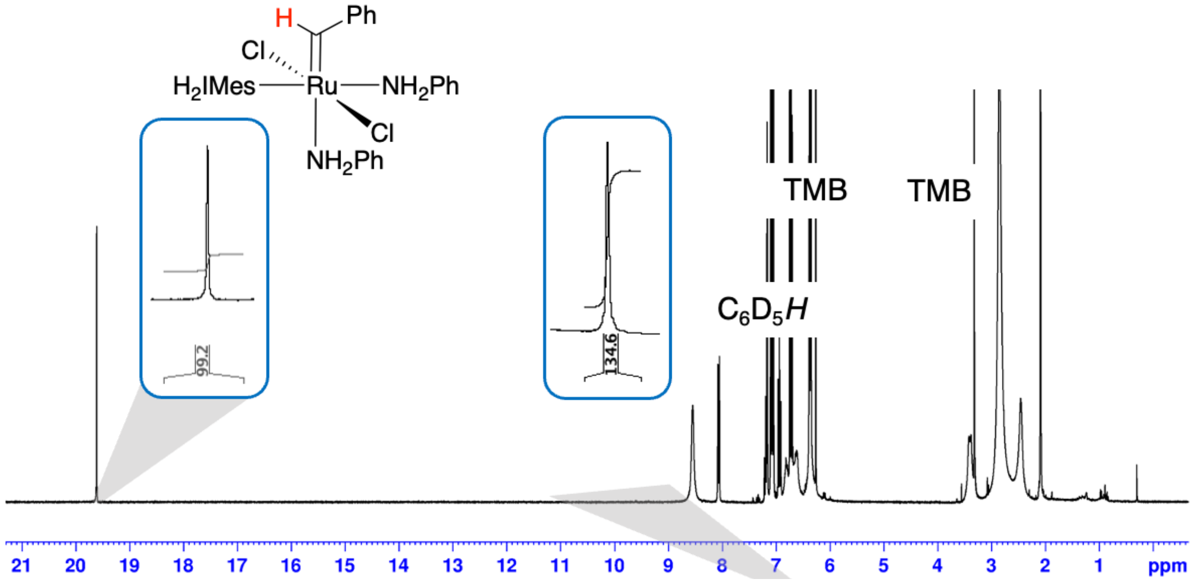

(Continues on next page)

(c) t = 24 h

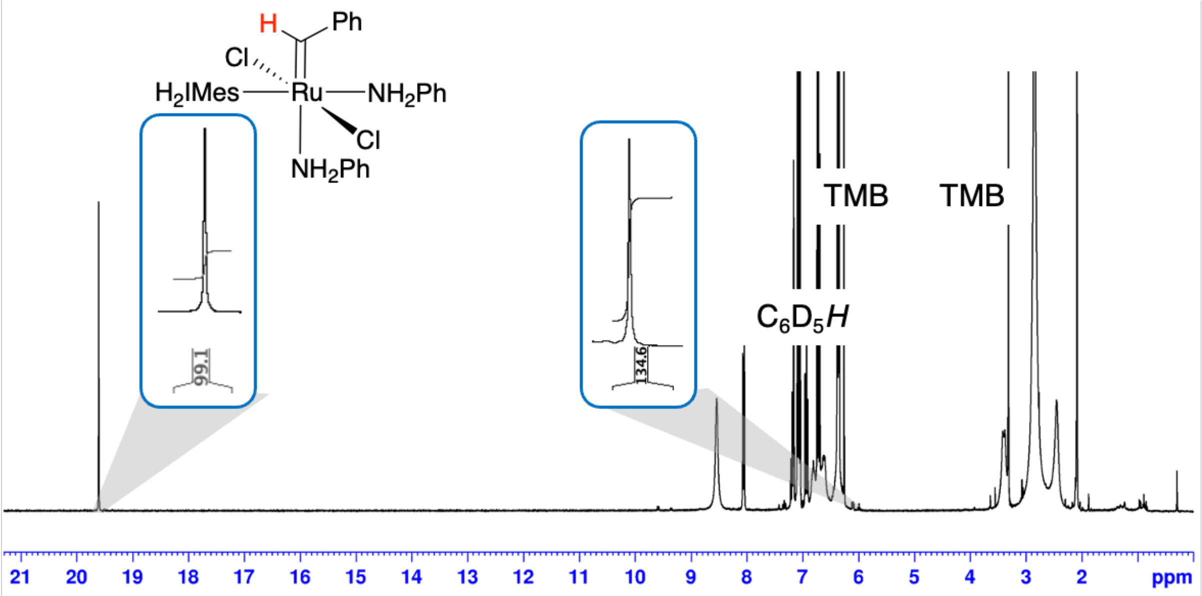

Figure S22. ^{1}H NMR spectra (300 MHz, C_6D_6) showing the reaction of **GIII** with NH_2Ph. (a) Before addition of NH_2Ph. (b) After adding NH_2Ph and mixing for 1 h. (c) After mixing for 24 h.

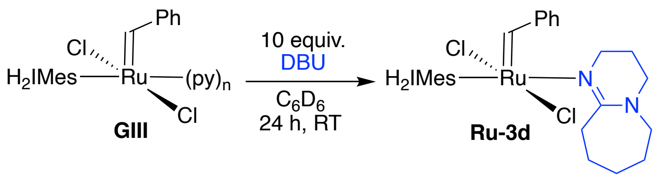

(a) Initial ratio of **GIII** to TMB internal standard, prior to adding amine

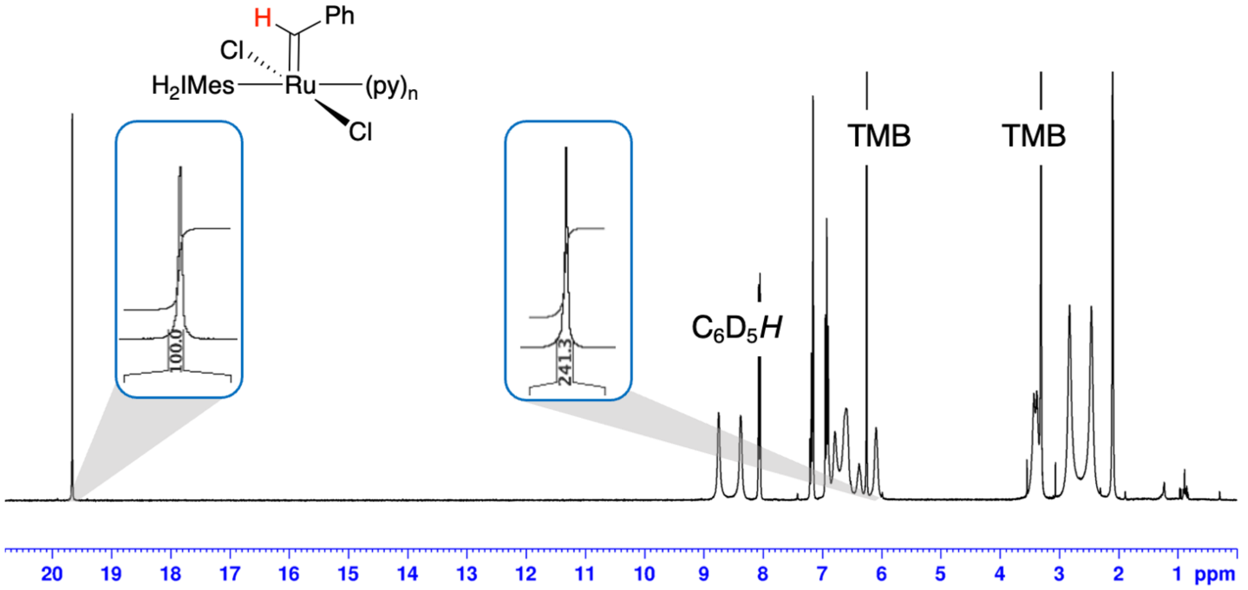

(b) t = 1 h

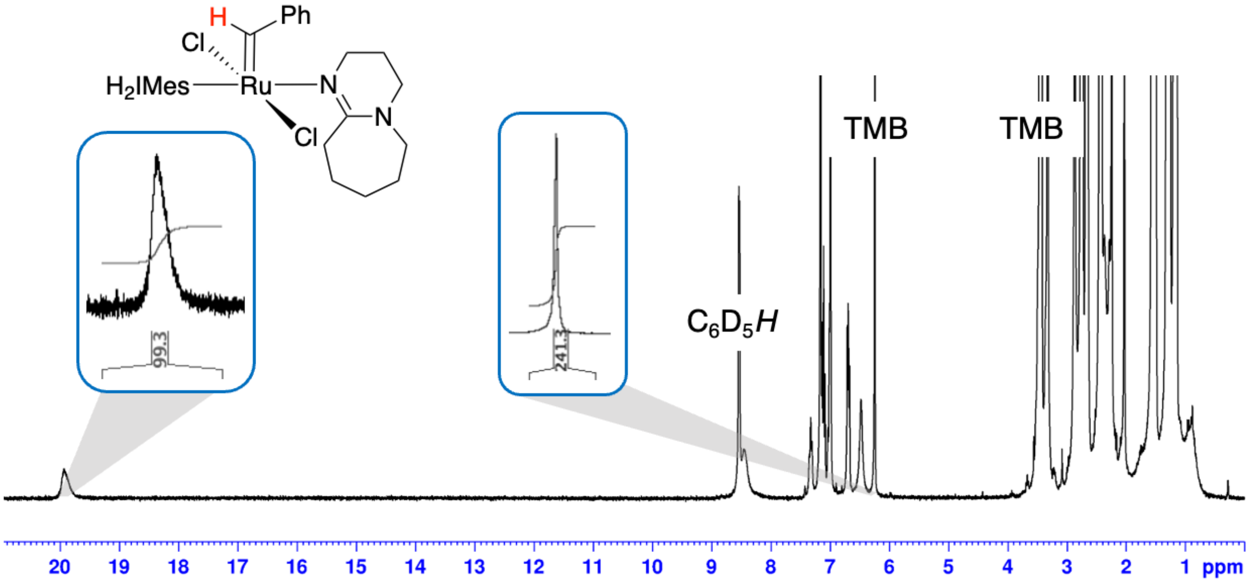

(Continues on next page)

(c) t = 24 h

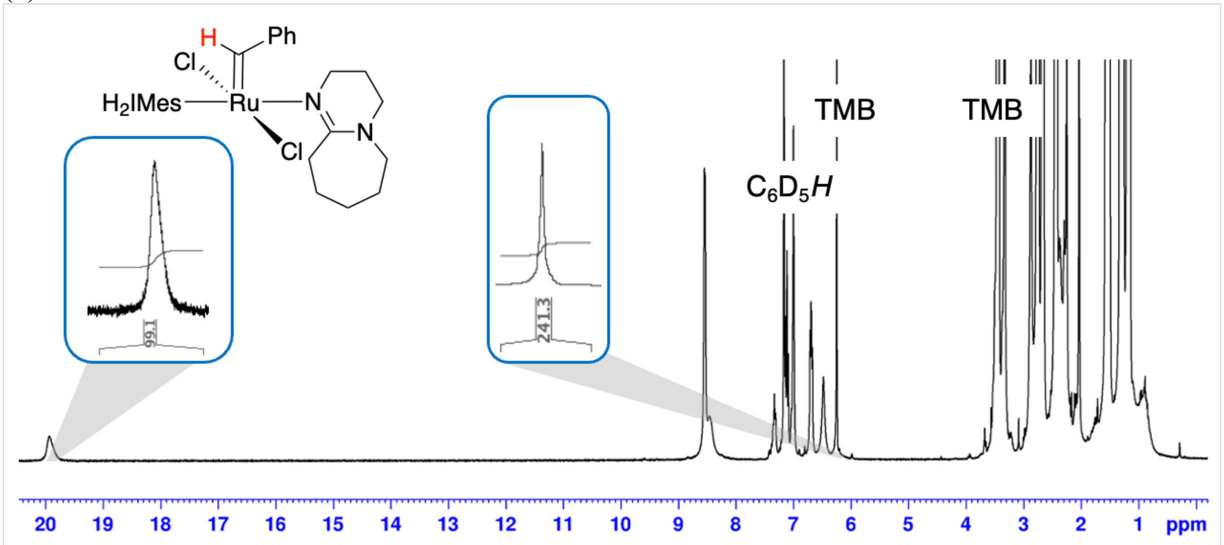

Figure S23. ^{1}H NMR spectra for reaction of **GIII** with DBU. (300 MHz, C_6D_6). (a) Before addition of DBU. (b) After adding DBU and mixing for 1 h. (c) After 24 h.

S3. GPC Chromatograms for Polymer Products.

Table S2. Summary of GPC Data

Polymer	Additives	Theoretical M_n (kg/mol)	Experimental M_n (kg/mol)	Đ
**poly(1)**	none	12.4	– [a]	– [a]
**poly(1)•HCl**	HCl	16.1	8.1[b]	– [a]
**poly(3-endo)**	none	25.4	125.5	1.27
	$NH_2{}^{n}Bu$	25.4	– [a]	– [a]
	HCl, $NH_2{}^{n}Bu$	25.4	94.8	1.42
**poly(3-exo)**	none	25.4	71.6	1.09
	$NH_2{}^{n}Bu$	25.4	– [a]	– [a]
	HCl, $NH_2{}^{n}Bu$	25.4	83.4	1.24
	NEt_3	25.4	81.4	1.11

[a] ROMP does not proceed. [b] Solubility precludes GPC analysis; assessed by endgroup analysis (^{1}H NMR).

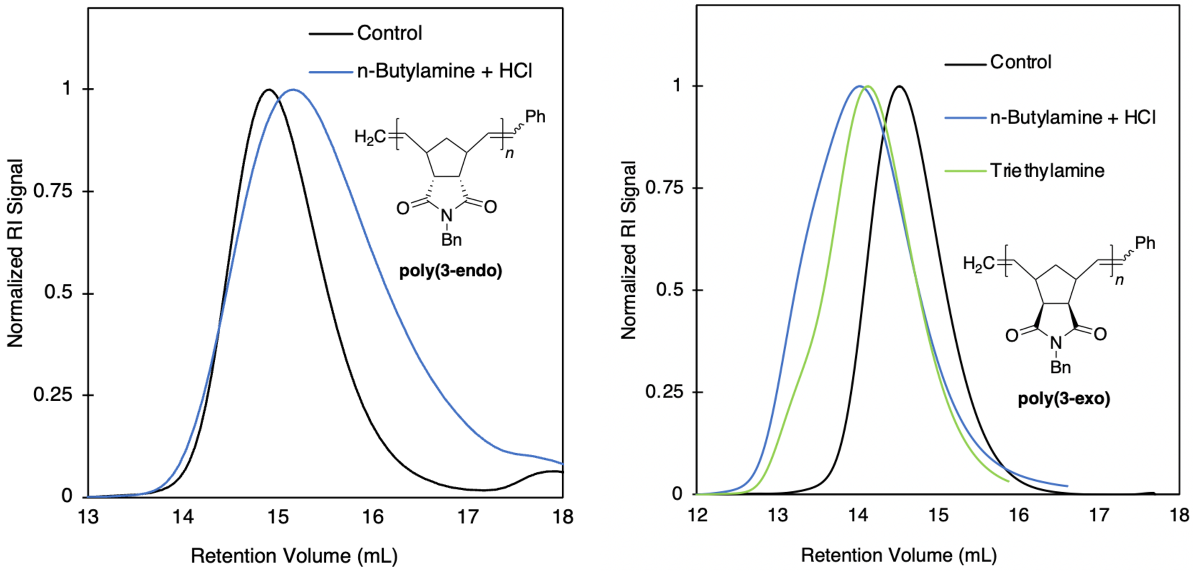

Figure S24. GPC traces for polymers prepared in the presence of various additives. Left: **poly(3-endo)**. Right: **poly(3-exo)**. Legend indicates additives present during ROMP. Control reactions have no additives present.

S4. Mass Spectrum

(a) Peak corresponding to [**Ru-6/7**–py–H]$^+$ in mass spectrum of the **Ru-3f**, **Ru-6**, **Ru-7** mixture.[a]

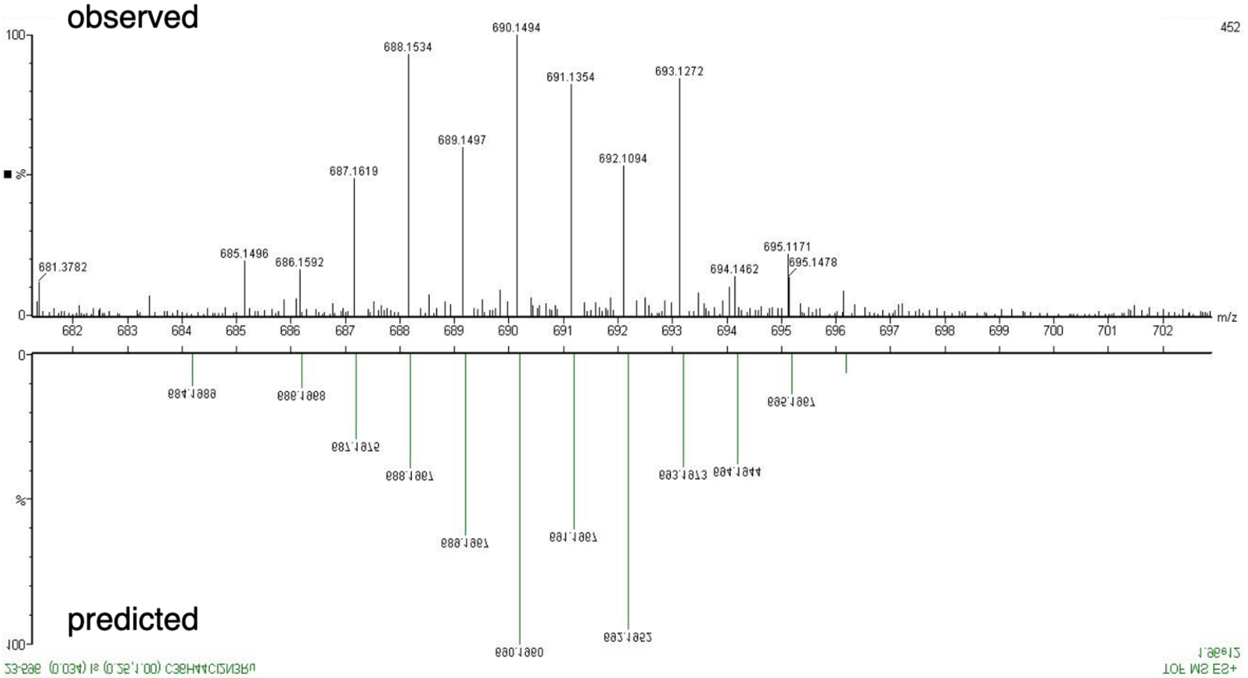

(b) Full spectrum.

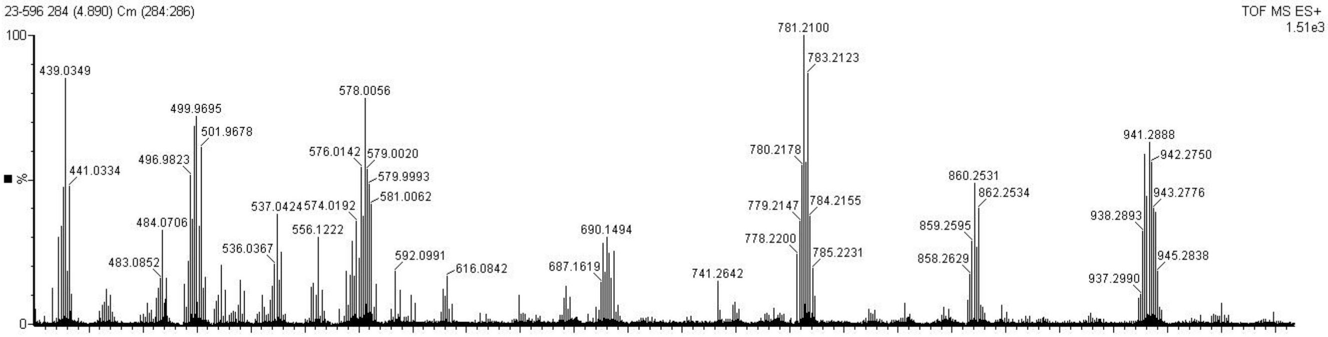

Figure S25. Mass spectrum of mixture containing **Ru-3f**, **Ru-6**, and **Ru-7**. (a) Expanded view of the isotope pattern of the peak at 690.14 m/z identified as [M-pyH]$^+$ (top) compared to the predicted isotope pattern (inverted). (b) Full spectrum.

[a]**Ru-3f**, **Ru-6**, and **Ru-7** have the same mass.

S5. References

(1) Sanford, M. S.; Love, J. A.; Grubbs, R. H., A Versatile Precursor for the Synthesis of New Ruthenium Olefin Metathesis Catalysts. *Organometallics* **2001**, *20*, 5314–5318.

(2) Camm, K. D.; Castro, N. M.; Liu, Y.; Czechura, P.; Snelgrove, J. L.; Fogg, D. E., Tandem ROMP-Hydrogenation with a Third-Beneration Grubbs Catalyst. *J. Am. Chem. Soc.* **2007**, *129*, 4168–4169.

(3) Bloembergen, N.; Purcell, E. M.; Pound, R. V., Relaxation Effects in Nuclear Magnetic Resonance Absorption. *Phys. Rev.* **1948**, *73*, 679-712.

(4) Blanco, C. O.; Fogg, D. E., Water-Accelerated Decomposition of Olefin Metathesis Catalysts. *ACS Catal.* **2023**, *13*, 1097–1102.

(5) Blacquiere, J. M.; Jurca, T.; Weiss, J.; Fogg, D. E., Time as a Dimension in High-Throughput Homogeneous Catalysis. *Adv. Synth. Catal.* **2008**, *350*, 2849–2855.

(6) Thomas, R. M.; Fedorov, A.; Keitz, B. K.; Grubbs, R. H., Thermally Stable, Latent Olefin Metathesis Catalysts. *Organometallics* **2011**, *30*, 6713–6717.

(7) Sanford, M. S.; Henling, L. M.; Grubbs, R. H., Synthesis and Reactivity of Neutral and Cationic Ruthenium(II) Tris(pyrazolyl)borate Alkylidenes. *Organometallics* **1998**, *17*, 5384–5389.

(8) Lummiss, J. A. M.; Ireland, B. J.; Sommers, J. M.; Fogg, D. E., Amine-Mediated Degradation in Olefin Metathesis Reactions that Employ the Second-Generation Grubbs Catalysts. *ChemCatChem* **2014**, *6*, 459–463.
